# Investigating the Active Substance and Mechanism of San-Jiu-Wei-Tai Granules via UPLC-QE-Orbitrap-MS and Network Pharmacology

**DOI:** 10.1155/2022/1487903

**Published:** 2022-10-17

**Authors:** Gengyuan Yu, Tonghua Zhang, Haoran Xu, Yuelin Bi, Xin Feng, Jiaqi Wang, Tianyi Li, Chenning Zhang, Yikun Sun

**Affiliations:** ^1^School of Chinese Materia Medica, Beijing University of Chinese Medicine, Beijing, China; ^2^Department of Pharmacy, Xiangyang No. 1 People's Hospital, Hubei University of Medicine, Xiangyang, China

## Abstract

San-Jiu-Wei-Tai granules (SJWTG) are a significant Chinese patent medicine for the treatment of chronic gastritis (CG), having outstanding advantages in long-term treatment; however, the chemical composition and potential mechanism have not been investigated until now. In this study, a rapid separation and identification method based on UPLC-QE-Orbitrap-MS was established, and 95 chemical components from SJWTGs were identified, including 6 chemical components of an unknown source that are not derived from the 8 herbs included in SJWTGs. The identified chemical components were subsequently analysed by network pharmacology, suggesting that the core targets for the treatment of CG with SJWTGs were EGFR, SRC, AKT1, HSP90AA1, MAPK1, and MAPK3 and thus indicating that SJWTGs could reduce the inflammatory response of gastric epithelial cells and prevent persistent chronic inflammation that induces cancerization by regulating the MAPK signalling pathway and the C-type lectin receptor signalling pathway as well as their upstream and downstream pathways in the treatment of CG. The key bioactive components in SJWTGs were identified as 2,6-bis(4-ethylphenyl)perhydro-1,3,5,7-tetraoxanaphth-4-ylethane-1,2-diol, a chemical component of an unknown source, murrangatin, meranzin hydrate, paeoniflorin, and albiflorin. The results of molecular docking showed the strong binding interaction between the key bioactive components and the core targets, demonstrating that the key bioactive components deserve to be further studied and considered as Q-markers. By acting on multiple targets, SJWTG is less susceptible to drug resistance during the long-term treatment of CG, indicating the advantage of Chinese patent medicines. Furthermore, the preventive effect of SJWTGs on gastric cancer also demonstrates the superiority of preventive treatment of disease with traditional Chinese medicine.

## 1. Introduction

Chronic gastritis (CG) is a disease involving chronic inflammatory or atrophic lesions of the gastric mucosa caused by various etiology [1], with clinical symptoms such as abdominal pain, fullness, burning sensation, acid reflux and belching, and loss of appetite as well as emotional changes like anxiety and depression. According to the new Sydney system for the classification of gastritis, CG is divided into chronic superficial gastritis (chronic non-atrophic gastritis) and chronic atrophic gastritis [2]. The incidence of CG is associated with a variety of factors, mainly including *Helicobacter pylori* (*H. pylori*) infection [3], duodenal-gastric reflux [4], drug factors [5], and autoimmunity [6]. *H. pylori* infection is the most common cause of CG, accounting for about 90%, and is also the main cause of gastric cancer [7]. However, the pathogenesis of CG has not been elucidated and remains to be further explored.

CG is the most common lifelong disease in human beings, possessing the characteristics of long duration and a strong chance of reoccurrence. It is estimated that more than half of the world's population suffers from CG, and millions of people worldwide die prematurely each year as a result of gastric cancer and gastric ulcer, which are the sequelae of CG [6]. In China, the prevalence of CG is the highest among digestive system diseases. In patients with long-lasting CG, the gastric epithelial cells are persistently infiltrated by inflammation, resulting in a variety of abnormal gene expressions and gene mutations [8]. Morphologically, prolonged inflammatory infiltration results in a thinning of the gastric mucosa, that is, gland atrophy, which indicates that chronic superficial gastritis has developed into chronic atrophic gastritis [2]. During the period of chronic atrophic gastritis, patients are so prone to gastric cancer that the World Health Organization has listed chronic atrophic gastritis as a pre-gastric cancer state [1]. Recently, research has shown that the pathogenesis of gastric cancer may be closely related to spasmolytic polypeptide-expressing metaplasia (SPEM). SPEM appears in the development of CG and represents the physiological healing response to injury [9], which also indicates that CG, as a pre-gastric cancer state, cannot be ignored. Nonetheless, most of the alterations can be reversed during the period of chronic superficial gastritis, suggesting that the timely and effective treatment of CG is of great significance.

Generally, Western medicine adopts triple (proton pump inhibitors, clarithromycin, and amoxicillin or metronidazole) or quadruple therapy (proton pump inhibitors, bismuth subcitrate or subsalicylate, tetracycline, and metronidazole) in treating CG. Nevertheless, there is still a risk of recurrence, drug resistance, and adverse reactions [10, 11]. In contrast, Chinese patent medicines have outstanding advantages in the long-term treatment of CG due to their “multi-component and multi-target” characteristics. Among them, San-Jiu-Wei-Tai granules (SJWTGs) have been recommended for the treatment of CG in the clinical application guidelines on Chinese patent medicines for the treatment of CG (2020) [12]. SJWTG includes 8 herbs, *Melicope pteleifolia* (Champ. ex Benth.) T. G. Hartley, *Murraya exotica* L., *Zanthoxylum nitidum* (Roxb.) DC., *Dolomiaea costus* (Falc.) Kasana and A. K. Pandey, *Scutellaria baicalensis* Georgi, *Poria cocos* (Schw.) Wolf, *Rehmannia glutinosa* (Gaertn.) DC., and *Paeonia lactiflora* Pall., and is effective in clearing heat and dampness, promoting Qi and activating blood, softening the liver, and relieving pain. The ethanol extract of SJWTGs has been shown to treat anti-gastric ulcers, inhibit mucosal erosion and bleeding, and prevent large mucosal necrosis and shedding [13], but the mechanism remains unclear. To date, the chemical constituents of SJWTGs have not been systematically isolated and identified, and there has been no pharmacological evaluation of SJWTGs in treating CG. According to the Chinese Pharmacopoeia 2020 Edition, only baicalin is adopted as the Q-marker of SJWTGs [14], which makes quality control inadequate. Therefore, it is urgent to investigate the chemical components of SJWTGs and explore the potential mechanism for treating CG.

**Figure 1:**
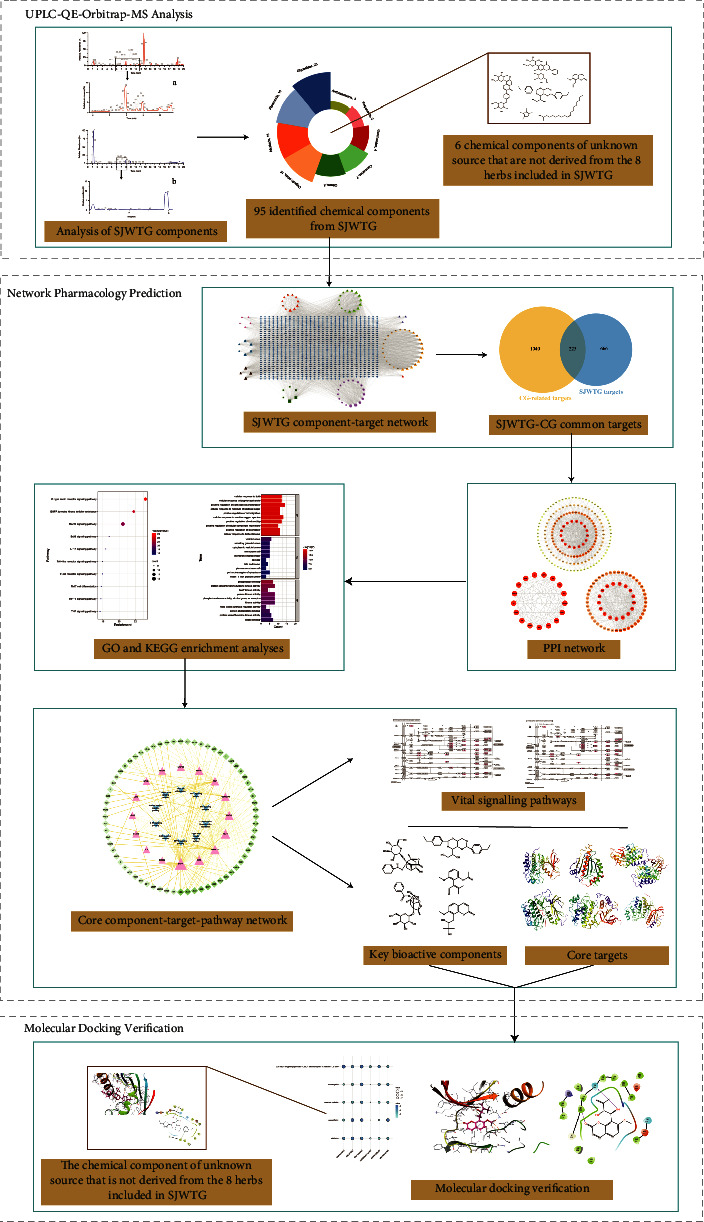
The workflow of this study.

Considering the “multi-component and multi-target” characteristics of traditional Chinese medicine (TCM), UPLC-QE-Orbitrap-MS was used to identify the chemical components in SJWTGs, due to its high resolution, high sensitivity, and high selectivity. Network pharmacology was then performed for insight into its potential mechanism in treating CG, including the key bioactive components, the core potential targets, and the vital signalling pathways. Finally, molecular docking was conducted to further verify the strong binding interactions between the key bioactive components and the core potential targets. The workflow of this study is illustrated in Figure 1. Our work lays a foundation for the further pharmacodynamics study of SJWTGs and guides clinical medication and quality control.

## 2. Materials and Methods

### 2.1. Materials and Reagents

SJWTG (Lot number: 2101078F); acetonitrile (MS grade), formic acid (MS grade), and methanol (MS grade) [all purchased from Thermo Fisher Scientific (China) Co., Ltd.]; and ultrapure water (ultra-purified by Milli-Q Advantage A10 Water system) were used.

### 2.2. Preparation of SJWTG Extract

Accurately weighed granules (1.00 g) were suspended in 10 mL 50% (*v*/*v*) methanol-water, ultrasonically extracted for 30 min, and then cooled to room temperature. After centrifuging at 10000 rpm for 10 min, 20 *µ*L supernatant was diluted 50 times with 50% (*v*/*v*) methanol-water, and the diluted sample was then centrifuged at 12000 rpm for 10 min to obtain the final sample solution.

### 2.3. Main Instruments

The following instruments were used: Thermo Scientific Q Exactive (Thermo Scientific); Xcalibur (Thermo Scientific); Vanquish Duo UHPLC System for Dual LC Workflows (Thermo Scientific); Waters BEH UPLC C18 column (1.7 *µ*m, 2.1 × 150 mm, Milford, MA, USA); CPA225D Electronic balance (SARTORIUS); KQ5200DA Ultrasonic Cleaners (Kunshan Shumei, China); Pico 17 High-speed centrifuge (Thermo Scientific); and Nitrogen evaporator N-EVAP 116 (Organomation).

### 2.4. UPLC-QE-Orbitrap-MS Conditions

We followed the methods of Feng et al. and the methods' description partly matches their wording [15]. Waters ACQUITY UPLC BEH C18 column (1.7 *µ*m, 2.1 × 150 mm, Milford, MA, USA), column temperature 40°C, injection volume 3 *µ*L, flow rate 0.3 mL/min, mobile phase comprising 0.1% formic acid aqueous solution (A) and acetonitrile (B). The eluting program was 5–5%B for 0–1 min, 5–95%B for 1–18 min, and 95–95%B for 18–20 min.

The ion source was electron spray ionization (ESI); MS was operated in negative/positive mode; the scan mode was full scan/ddMS2, with positive and negative ion alternating scanning; and the scanning mode was 100–1300 Da, with a capillary temperature of 350°C. The spray voltage in positive mode was 3800 V, the spray voltage in negative mode was 3200 V, the sheath gas was 35 arb, and the auxiliary gas was 15 arb. Three collision energies of low, medium, and high were used for MS2. The positive ion mode was 20 V, 40 V, and 60 V, and the negative ion mode was 30 V, 50 V, and 70 V. Resolutions of full scan and ddMS2 were 70000 FWHM (full width at half maximum) and 17500 FWHM, respectively.

### 2.5. Compound Identification

The chemical components in the SJWTG extract were identified according to the chromatographic retention time, relative molecular mass, fragment ion information, relevant literature data, and MS fragmentation ion.

### 2.6. Network Pharmacology Analyses

#### 2.6.1. SJWTG Targets

The 95 identified compounds were selected as target chemical components, the targets of which were obtained by Swiss Target Prediction (https://www.swisstargetprediction.ch/) with the conditions set as “*Homo sapiens*” and probability value >0. Afterwards, the SJWTG component-target network was constructed by Cytoscape 3.7.2 software.

#### 2.6.2. CG-Related Targets

CG-related targets were retrieved from Online Mendelian Inheritance in Man (OMIM, https://omim.org/) and GeneCards (https://www.genecards.org/) with “chronic gastritis” as the keywords.

#### 2.6.3. SJWTG-CG Common Targets

SJWTG-CG common targets were obtained by taking the intersection of SJWTG targets and CG-related targets via a Venn diagram plotted by EHBIO (https://www.ehbio.com/).

#### 2.6.4. PPI Network and Evaluation

The SJWTG-CG common targets were imported into the STRING database (https://string-db.org/cgi/input.pl) to obtain protein-protein interaction (PPI) information. The confidence score was set to 0.9 or higher, and the edge free proteins were excluded in order to ensure the reliability of the research data. Cytoscape 3.7.2 software was used to visualize the structure of the protein network and analyse its topological characteristics. The core targets for the treatment of CG with SJWTGs were obtained by taking the median of degree centrality (DC), betweenness centrality (BC), and closeness centrality (CC), as screening conditions, twice.

#### 2.6.5. GO Analysis and KEGG Enrichment Analyses

The core targets for the treatment of CG with SJWTGs were imported into Metascape (https://metascape.org/) for gene ontology (GO) and Kyoto Encyclopedia of Genes and Genomes (KEGG) analyses, and the enrichment diagram was plotted by bioinformatics.

#### 2.6.6. Core Component-Target-Pathway Network

The effective components, core targets, and action pathways of SJWTGs were imported into Cytoscape 3.7.2 software to establish the core component-target-pathway network, with the purpose of mining the key bioactive components and vital pathways. KEGG Mapper (https://www.kegg.jp/kegg/mapper/) was then used to annotate the roles of important targets of the vital pathways.

### 2.7. Molecular Docking

The two-dimensional (2D) structures of the compounds used as ligands were obtained from the PubChem database (https://pubchem.ncbi.nlm.nih.gov/), processed to minimize energy, and transformed into the MOL format as ligands for docking through Chem3D. The X-ray crystal structures of the proteins were obtained from the Protein Data Bank (PDB, https://www.rcsb.org/). Afterwards, the structures of these proteins were optimized by Maestro 11.8 software, including the assignment of bonds and bond orders, the addition of hydrogens, filling in missing loops or side chains, capping uncapped termini, adjusting bonds and formal charges for metals, correcting mislabelled elements, and deleting unnecessary parts in the structure. Subsequently, each grid box was centred on the original ligand in each protein. The docking results were visualized and displayed as three-dimensional (3D) diagrams and 2D diagrams using Maestro 11.8.

## 3. Results

### 3.1. Analysis of SJWTG Components

#### 3.1.1. Chemical Component Analysis

**Figure 2:**
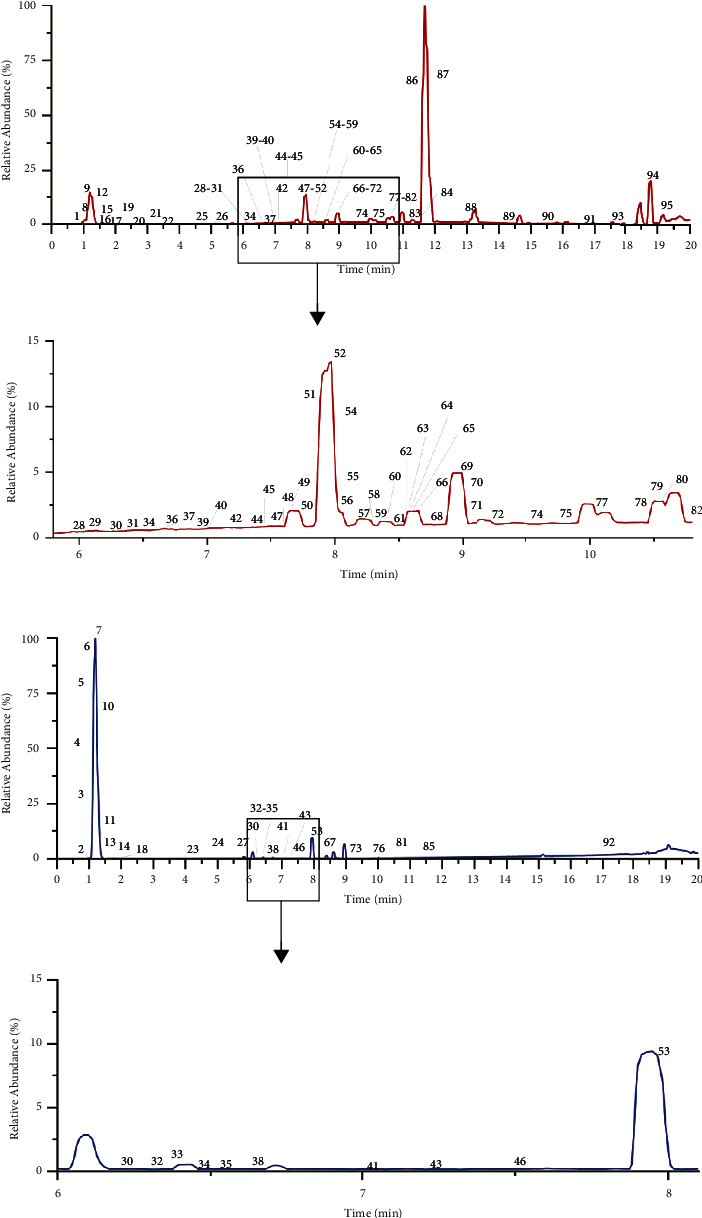
Base peak ion flow diagram of SJWTG samples under positive ion (a) and negative ion (b) by UPLC-QE-Orbitrap-MS.

**Figure 3:**
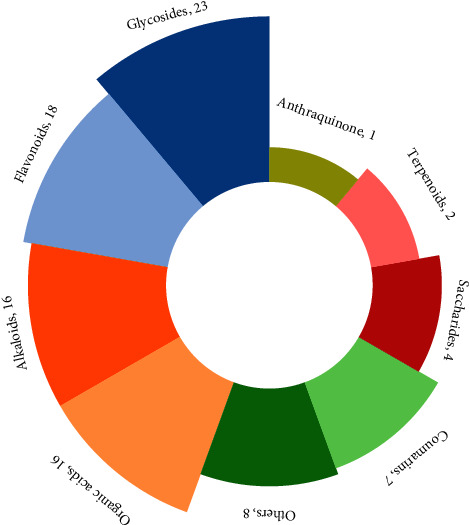
Classification of the 95 identified chemical components.

The retention time and mass spectrometry information of the compounds from SJWTGs were obtained by UPLC-QE-Orbitrap-MS. Due to the complexity of chemical components, the SJWTG samples were fully scanned under positive and negative ionization modes. The base peak intensity chromatogram (BPI) is shown in Figure 2. Based on accurate mass spectrometry information, such as fragment ions, and reference literature, 95 chemical components were identified in SJWTGs (Table 1) and were categorized into 9 classes of natural compounds (Figure 3). The chemical structures of 95 identified compounds are shown in Supplementary Materials (Figure S1).

#### 3.1.2. Identification of Main Compounds in SJWTGs

*(1) Identification of Characteristic Compounds in Melicope pteleifolia*. Compound 60 was detected at the retention time of 8.40 min and exhibited the quasi-molecular ion at m/z 247.0963 [M+H]^+^. Attributed to the elimination of H_2_O, the fragment ion at m/z 229.0858 [M−H_2_O+H]^+^ further fragmented in two ways: the first produced fragment ions at m/z 213.0546 [M−H_2_O−CH_4_+H]^+^ via the neutral loss of CH_4_, while the second fragment ion at m/z 175.0390 [M−H_2_O−C_4_H_6_+H]^+^ was the result of the successive elimination of C_4_H_6_, which was the dominant peak in the MS2 spectrum, indicating that the elimination of C_4_H_6_ might be the major fragmentation pathway. The subsequent fragment ion at m/z 147.0435 [M−H_2_O−C_4_H_6_−CO+H]^+^ corresponded to the loss of CO, and the consecutive loss of CO and CO led to the formation of a product ion at m/z 213.0546 [M−H_2_O−C_4_H_6_−3CO+H]^+^. Using the data in MassBank of North America (https://mona.fiehnlab.ucdavis.edu/), compound 60 was identified as marmesin (Figure S2).

*(2) Identification of Characteristic Compounds in Murraya exotica*. Compound 72 was eluted at 9.15 min, with the protonated molecule ion at m/z 259.0962 [M+H]^+^. The fragment ion at m/z 231.1014 [M−CO+H]^+^ was formed by the successive elimination of CO. The fragment ion at m/z 203.0699 [M−2CO+H]^+^ was observed due to the characteristic coumarin loss of CO, and the subsequent loss of CH_2_ led to the fragment ions at m/z 189.0545 [M−2CO−CH_2_+H]^+^. The fragment ion at m/z 159.0440 [M−2CO−CH_2_−2CH_3_+H]^+^ was attributed to the consecutive loss of CH_3_ and CH_3_ and the high abundance ion at m/z 131.0492 [M−2CO−CH_2_−2CH_3_−CO+H]^+^ was caused by the loss of CO, producing the subsequent ion at m/z 103.0546 [M−2CO−CH_2_−2CH_3_−2CO+H]^+^ via the elimination of CO. Using MassBank of North America, the main fragment ions were found to be identical to those of murralongin (Figure S3).

*(3) Identification of Characteristic Compounds in Zanthoxylum nitidum*. Compound 61 yielded the molecule ion at m/z 348.1227 [M]^+^ at the retention time of 8.50 min. MS2 spectra of this compound exhibited the neutral loss of CH_4_, resulting in a product ion at m/z 332.0913 [M−CH_4_]^+^. The fragment ion at m/z 332.0931 further fragmented in two ways, both of which broke up into the ion at m/z 290.0811 [M−CH_4_−CH_2_−CO]^+^, corresponding to the consecutive loss of CH_2_ and CO: the first pathway exhibited a loss of CH_2_ first, leading to the fragment ion at m/z 318.0760 [M−CH_4_−CH_2_]^+^, while the second exhibited a loss of CO first, leading to the fragment ion at m/z 304.0964 [M−CH_4_−CO]^+^. Based on these results and the reference standard [16], compound 61 was identified as nitidine (Figure S4).

*(4) Identification of Characteristic Compounds in Dolomiaea costus*. Compound 75 eluted at 9.83 min and exhibited the quasi-molecular ion at m/z 233.1534 [M+H]^+^. The product ion m/z 215.1428 [M−H_2_O+H]^+^ was observed due to the neutral loss of H_2_O, and the MS2 spectra showed a high abundance ion at m/z 187.1480 [M−H_2_O−CO+H]^+^, resulting from the successive elimination of CO; the removal of H_2_O followed by CO is a significant characteristic of the cleavage of the lactone ring. The fragment ions at m/z 145.1012 [M−H_2_O−CO−C_3_H_6_+H]^+^, m/z 145.1012 [M−H_2_O−CO−C_3_H_6_−CH_2_+H]^+^, and m/z 145.1012 [M−H_2_O−CO−C_3_H_6_−CH_2_−C_2_H_2_+H]^+^ were attributed to the consecutive loss of C_3_H_6_, CH_2_, and C_2_H_2_, indicating that compound 75 broke up into a chain compound of conjugated polyene after the consecutive loss of H_2_O and CO. Thus, compound 75 was identified as costunolide by consulting MassBank Europe (https://massbank.eu/MassBank/) (Figure S5).

*(5) Identification of Characteristic Compounds in Scutellaria baicalensis*. Compound 34 showed the quasi-molecular ion at m/z 463.0872 [M+H]^+^ at the retention time of 6.49 min. The neutral loss of glucuronic acid is an important characteristic of flavonoid glycoside since the O-glucosylic bond is easily cleaved to generate aglycone, corresponding to the fragment ion at m/z 287.0548 [M−C_6_H_8_O_6_+H]^+^. Besides, the fragment of glucuronic acid exhibited the ion at m/z 141.0185 [M−C_15_H_14_O_8_+H]^+^, suggesting that compound 34 is a glucuronoside. With regard to the fragmentation of aglycone, the fragment ions at m/z 169.0137 [M−C_15_H_14_O_8_−C_8_H_6_O+H]^+^ and 119.0494 [M−C_15_H_14_O_8_−C_7_H_4_O_5_+H]^+^ were attributed to retro-Diels–Alder (RDA) reaction, which is the characteristic of the flavone fragmentation pathway. Furthermore, the ion at m/z 169.0137 showed that the three substituent OH groups were located on the A-ring, while the ion at m/z 119.0494 showed that a substituent OH group was located on the B-ring. The fragment ion at m/z 123.0078 [M−C_15_H_14_O_8_−C_8_H_6_O−CO−H_2_O+H]^+^ was formed by the consecutive loss of CO and H_2_O from the ion at m/z 169.0137. Moreover, the fragment ion at m/z 91.0547 [M−C_15_H_14_O_8_−C_7_H_4_O_5_−CO+H]^+^ was observed due to the loss of CO from the ion at m/z 119.0494. Using the data in mzCloud (https://www.mzcloud.org/), compound 34 was identified as scutellarin (Figure S6).

*(6) Identification of Characteristic Compounds in Poria cocos*. Compound 8 exhibited the protonated molecule ion at m/z 152.0567 [M+H]^+^, which eluted at 1.22 min. The parent ion further fragmented via three competing fragmentation pathways: the first through the consecutive loss of CN, NH_2_, and NH_3_ to produce fragment ions at m/z 110.0351 [M−CN−NH_2_+H]^+^ and 93.0578 [M−CN−NH_2_−NH_3_+H]^+^; the second via the consecutive loss of CONH and CN to give fragment ions at m/z 109.517 [M−CONH+H]^+^ and 83.406 [M−CONH−CN+H]^+^; and the third by the cleavage of the pyrimidine ring, resulting in the fragment ion at m/z 135.0301 [M−NH_3_+H]^+^ by the neutral loss of NH_3_. As the MS2 spectra of this compound exhibited a high relative abundance of ions at m/z 110.0351 and 93.0578, the first fragmentation pathway was shown to be dominant, which is the characteristic of the cleavage of purine. Moreover, the fragment ions at m/z 110.0351, 109.517, and 135.0301 suggested that the substituent groups of CO and NH_2_ were located in the pyrimidine ring. Therefore, compound 8 was identified as guanine by consulting mzCloud (Figure S7).

*(7) Identification of Characteristic Compounds in Rehmannia glutinosa*. Compound 35 showed an [M−H]^−^ peak at m/z 623.1966 at the retention time of 6.51 min. Due to the cleavage of the O-glucosylic bond linked with the carbonyl group, the parent ion produced fragment ions at m/z 461.1667 [M−C_9_H_6_O_3_−H]^−^ and 161.0238 [M−C_20_H_30_O_12_−H]^−^. The ion at m/z 135.0443 [M−C_9_H_6_O_3_−C_12_H_22_O_10_−H]^−^ was formed by the loss of C_12_H_22_O_10_ from the ion at m/z 461.1667, indicating that compound 35 is a diglucoside with two glucosylic bonds between glycoside and aglycone. The fragment ion at m/z 133.0287 [M−C_20_H_30_O_12_−CO−H]^−^ was attributed to the elimination of CO from the ion at m/z 161.0238. The parent ion also exhibited an ion at m/z 179.0347 [M−C_20_H_28_O_11_−H]^−^, which was unstable and tended to lose H_2_O, leading to the fragment ion at m/z 161.0238 [M−C_20_H_28_O_11_−H_2_O−H]^−^. According to mzCloud, compound 35 was identified as acteoside (Figure S8).

*(8) Identification of Characteristic Compounds in Paeonia lactiflora*. Compound 26 was eluted at 5.78 min and displayed the protonated molecule ion at m/z 481.1707 [M+H]^+^. Cleavage of the O-glucosylic bond resulted in the parent ion breaking up into aglycone and glycose, subsequently exhibiting fragment ions at m/z 197.0808 [M−C_13_H_16_O_7_+H]^+^ and 105.0338 [M−C_16_H_24_O_10_+H]^+^, corresponding to the cleavage of the ester bond. The consecutive loss of H_2_O and CO led to the formation of product ions at m/z 179.0701 [M−C_13_H_16_O_7_−H_2_O+H]^+^ and 151.0754 [M−C_13_H_16_O_7_−H_2_O−CO+H]^+^, indicating the existence of a lactone ring. The fragment ion at m/z 133.0648 [M−C_13_H_16_O_7_−H_2_O−CO−H_2_O+H]^+^ was attributed to the successive elimination of H_2_O. The high relative abundance ion at m/z 105.0388 was observed due to the stabilization of the aromatic hydrocarbon compared with the bridged-ring compound with the lactone ring. Using the data in mzCloud, compound 26 was identified as albiflorin (Figure S9).

*(9) Identification of Compounds of an Unknown Source*. Compound 88 was detected at the retention time of 13.16 min and exhibited the quasi-molecular ion at m/z 415.2113 [M+H]^+^. The parent ion further fragmented via three pathways: the first produced fragment ions at m/z 133.0649 [M−C_15_H_22_O_5_+H]^+^ by the loss of C_15_H_22_O_5_; the second produced the ion at m/z 119.0856 [M−C_15_H_20_O_6_+H]^+^ as a result of the successive elimination of C_15_H_20_O_6_, which was the dominant peak in the MS2 spectrum, indicating that the elimination of C_15_H_20_O_6_ might be the major fragmentation pathway; and the third exhibited the fragment ion at m/z 117.0700 [M−C_15_H_22_O_6_+H]^+^ corresponding to the elimination of C_15_H_22_O_6_ and subsequently produced the ion at m/z 103.0544 [M−C_15_H_22_O_6_−CH_2_+H]^+^ via the loss of CH_2_. Both of the ions at m/z 119.0856 and 117.0700 broke up into the fragment ion at m/z 91.0546 [M−C_17_H_24_O_6_+H]^+^ by the loss of C_2_H_4_ and C_2_H_2_, respectively. Based on these results, compound 88 was identified as 2,6-bis(4-ethylphenyl)perhydro-1,3,5,7-tetraoxanaphth-4-ylethane-1,2-diol by consulting mzCloud and is of an unknown source, not derived from the 8 herbs included in SJWTGs (Figure S10).

### 3.2. Network Pharmacology

#### 3.2.1. Construction of SJWTG Component-Target Network

**Figure 4:**
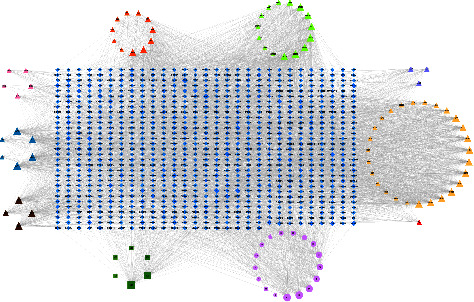
SJWTG component-target network. The diamond nodes stand for targets. The triangular nodes and the ellipse nodes stand for unique and common components of the 8 herbs included in SJWTGs, respectively. The rectangle nodes stand for chemical components of an unknown source. The brown triangular nodes, the blue triangular nodes, the green triangular nodes, the purple triangular nodes, the yellow triangular nodes, the red triangular node, and the orange triangular nodes stand for unique components of *Melicope pteleifolia*, *Murraya exotica*, *Zanthoxylum nitidum*, *Dolomiaea costus*, *Scutellaria baicalensis*, *Poria cocos*, *Rehmannia glutinosa* and *Paeonia lactiflora* respectively. The colour scales and the size of the nodes represent the degree level of components and targets.

The 95 identified compounds were selected as target chemical components to predict the potential mechanism of SJWTGs in treating CG by network pharmacology. Using the Swiss Target Prediction to predict targets, a total of 891 targets were obtained. The SJWTG component-target network was constructed by Cytoscape 3.7.2 software, including 985 nodes and 4956 edges (Figure 4). The colour scales and the size of the nodes represent the degree level of the components and targets. Among these nodes, 780 targets were from the unique components of the 8 herbs, 372 targets were from the common components, and 202 targets were from chemical components of an unknown source. Considering the aesthetic of the networks in this part, abbreviated names were given to the 95 identified compounds, as shown in the Supplementary Materials (Table S1).

#### 3.2.2. Acquisition of SJWTG-CG Common Targets

**Figure 5:**
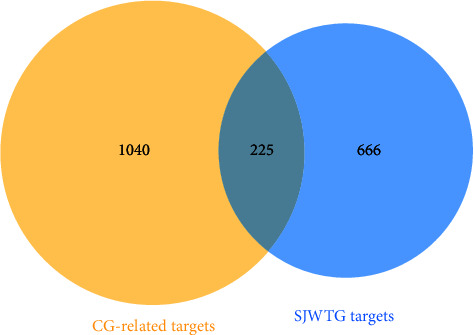
The Venn diagram of SJWTG-CG common targets.

Using the keyword “chronic gastritis” to search for disease targets in OMIM and GeneCards, and screening and merging to remove duplicates, a total of 1265 CG-related targets were obtained. A Venn diagram was plotted by EHBIO, and 225 SJWTG-CG common targets were obtained (Figure 5).

#### 3.2.3. Construction and Evaluation of PPI Network

**Figure 6:**
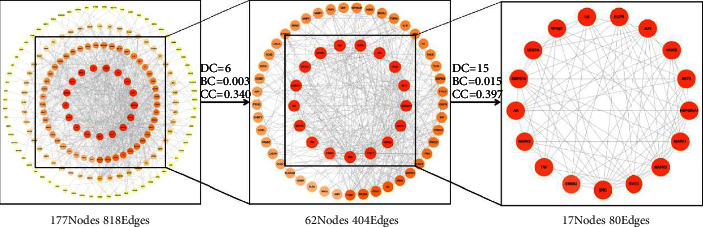
The PPI network of 225 common targets. The colour scales and the size of the circle represent the degree level of the target proteins.

The STRING database was employed to predict the PPIs of the 225 common targets. In this study, medium confidence >0.9 was set, edge free proteins were excluded, and the reliability of the research data was ensured. The topological characteristics of the protein network structure were analysed by Cytoscape 3.7.2 software, and 17 core targets for the treatment of CG with SJWTGs were screened, including SRC, STAT3, MAPK3, HSP90AA1, MAPK1, and AKT1 (Figure 6 and Table S2). The colour scales and the size of the circles represent the degree level of these target proteins.

#### 3.2.4. GO and KEGG Enrichment Analyses

**Figure 7:**
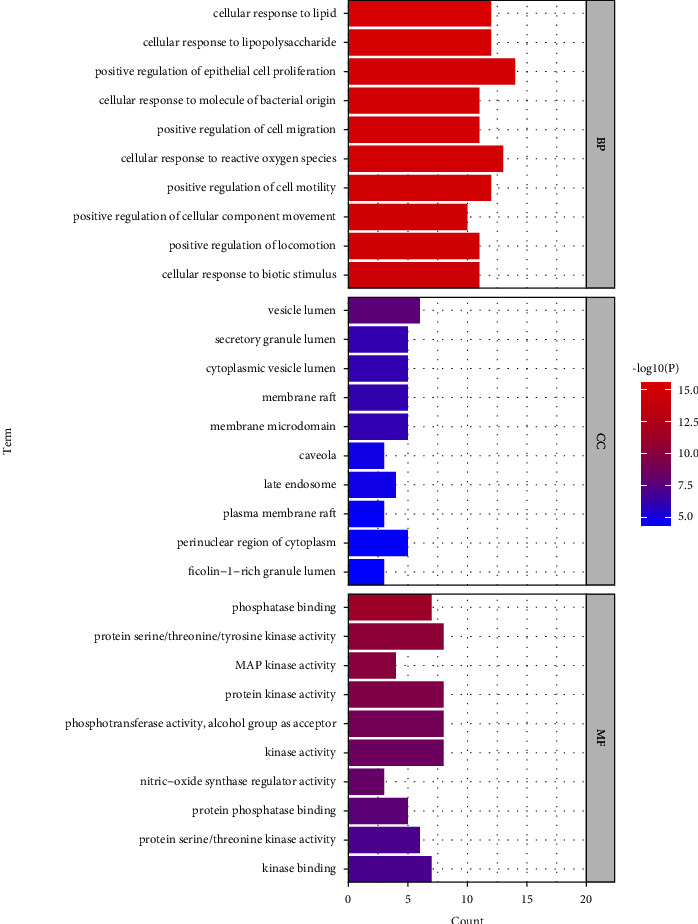
The GO enrichment analysis of core targets. The colour scales indicate the different thresholds for the *p* values.

To further investigate the core targets of SJWTGs in treating CG, GO analysis was carried out using Metascape, and biological process (BP), molecular function (MF), and cellular components (CCs) were screened out. The drawing was made using bioinformatics (Figure 7). The top 10 in the BP analysis are relevant to the cellular response to stimulus and the positive regulation of cell motility and migration, the top 10 in the CC analysis are related to the biomembrane system, and the top 10 in the MF analysis reveal that the core targets could affect the activity and binding of various phosphatases and kinases.

**Figure 8:**
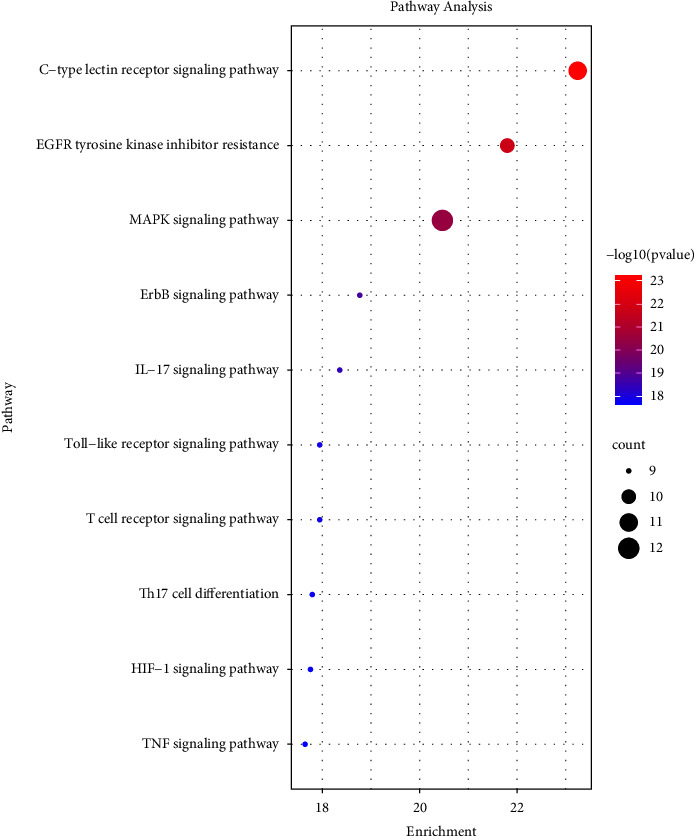
The KEGG pathway enrichment analysis of core targets. The colour scales indicate the different thresholds for the *p* values, and the sizes of the dots represent the number of genes corresponding to each term.

Metascape was employed to explore the KEGG pathway information that common targets may participate in. As a result, a total of 139 signal pathways were identified, and the top 10 pathways including distinctive genes were selected (Figure 8). The mechanism of SJWTGs in treating CG mainly involves the “C-type lectin receptor signalling pathway,” “EGFR tyrosine kinase inhibitor resistance,” and “MAPK signalling pathway.” The results of KEGG pathway analyses correspond to the results of the GO pathway analyses, indicating that the core targets of SJWTGs are related to the cellular response to stimulus by affecting the activity and binding of various phosphatases and kinases, which might be consistent with the treatment of CG with SJWTGs.

#### 3.2.5. Construction of Core Component-Target-Pathway Network

**Figure 9:**
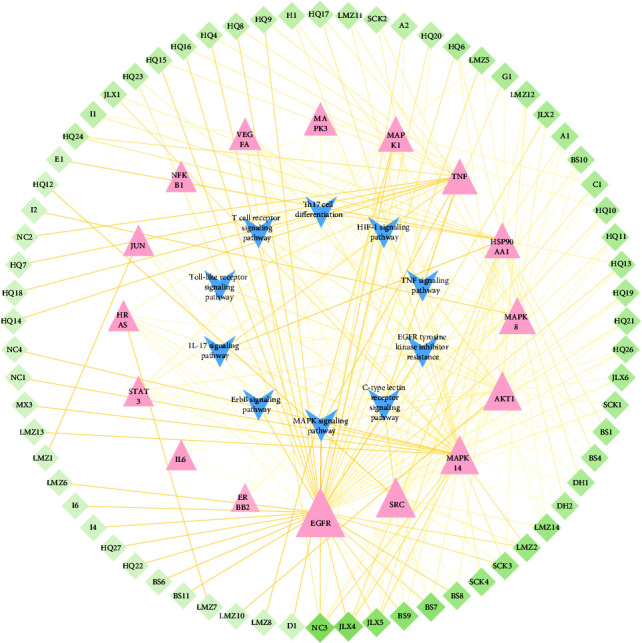
Core component-target-pathway network of SJWTGs. The diamond nodes stand for chemical components in SJWTGs. The triangular nodes stand for the core targets for the treatment of CG with SJWTGs. The V nodes stand for 10 signalling pathways. The colour scales and the size of the nodes represent the degree level of components, targets, and pathways. The colour scales of edges represent the edge betweenness.

**Figure 10:**
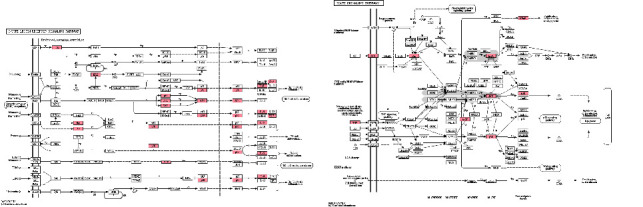
C-type lectin receptor signalling pathway (a) and MAPK signalling pathway (b). The pink rectangles represent the targets related to core genes in the component-target-pathway network.

With the purpose of gaining a holistic understanding of the underlying mechanism of SJWTGs, a component-target-pathway network was constructed by Cytoscape 3.7.2 software, including 94 nodes and 255 edges. Overall, 56 unique components of the 8 herbs, 13 common components, and 4 components of an unknown source participate in the regulation of 16 core targets and 10 pathways. The colour scales and the size of the nodes represent the degree level of the components, targets, and pathways. As shown in Figure 9, 2,6-bis(4-ethylphenyl)perhydro-1,3,5,7-tetraoxanaphth-4-ylethane-1,2-diol (NC3), murrangatin (JLX4), meranzin hydrate (JLX5), paeoniflorin (BS9), and albiflorin (BS7) are of great importance in treating CG. The top 10 essential targets in the component-target-pathway network are EGFR, SRC, MAPK14, AKT1, MAPK8, HSP90AA1, TNF, MAPK1, MAPK3, and VEGFA. Meanwhile, SRC, STAT3, MAPK3, HSP90AA1, MAPK1, AKT1, HRAS, EGFR, JUN, and IL6 are also vital targets in the PPI network. The duplicated targets between these two networks are EGFR, SRC, AKT1, HSP90AA1, MAPK1, and MAPK3, which are the core targets of SJWTGs in treating CG. Subsequently, we found that all of these targets play important roles in the MAPK signalling pathway and the C-type lectin receptor signalling pathway, suggesting that these two pathways are vital signalling pathways during the process by which SJWTG treats CG. KEGG Mapper was used to annotate the roles of the important targets of the two vital signalling pathways (Figure 10).

### 3.3. Molecular Docking

Based on the results of network pharmacology predictions, we believed that 2,6-bis(4-ethylphenyl)perhydro-1,3,5,7-tetraoxanaphth-4-ylethane-1,2-diol, murrangatin, meranzin hydrate, paeoniflorin, and albiflorin were the key bioactive components, while EGFR, SRC, AKT1, HSP90AA1, MAPK1, and MAPK3 were the core targets. These proteins not only play an important role in the KEGG signalling pathways but also serve as the key nodes of the PPI network and core component-target-pathway network. Therefore, the key bioactive components were used as ligands, and the core targets were used as proteins for the molecular docking, in order to further verify the network pharmacology results.

**Figure 11:**
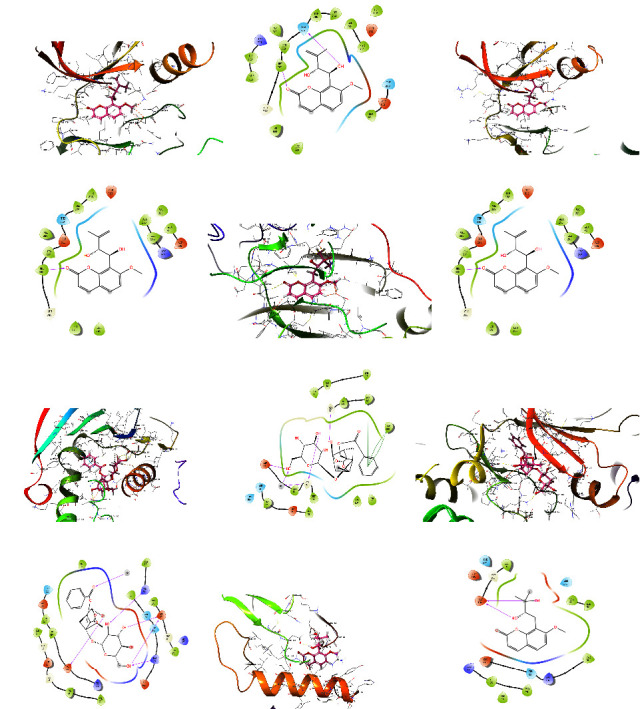
The 3D and 2D action mode graph of the representative compounds and related proteins. (a and b) The 3D and 2D action mode of murrangatin and EGFR (1M17). (c and d) The 3D and 2D action mode of murrangatin and SRC (1YOL). (e and f) The 3D and 2D action mode of meranzin hydrate and AKT1 (3QKK). (g and h) The 3D and 2D action mode of paeoniflorin and HSP90AA1 (1UYM). (i and j) The 3D and 2D action mode of paeoniflorin and MAPK1 (6SLG). (k and l) The 3D and 2D action mode of meranzin hydrate and MAPK3 (4QTB).

**Figure 12:**
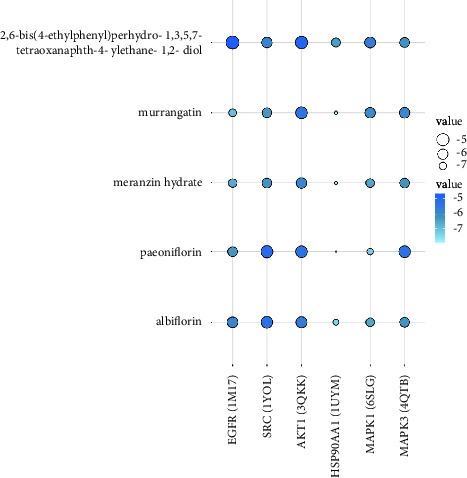
The heat map of molecular docking results. The smaller the dot and the lighter the colour, the more closely the compounds bind to the proteins.

As shown in Table 2, the binding energies were computed to evaluate the binding affinities of the 5 compounds with the 6 proteins, respectively. It is commonly recognized that a more negative binding energy value indicates a stronger binding affinity or a greater binding constant for the formation of the ligand-protein complex. The binding energies of the 5 compounds with the 6 proteins ranged from −4.698 to −7.876 kcal/mol, suggesting a degree of confidence in the network pharmacology results, namely that these 5 compounds may have potential therapeutic effects on CG by binding to the 6 proteins. Among them, EGFR and SRC have the lowest binding energy with murrangatin; AKT1 and MAPK3 have the lowest binding energy with meranzin hydrate; and HSP90AA1 and MAPK1 have the lowest binding energy with paeoniflorin. The 3D and 2D action mode graph of the representative compounds and related proteins are shown in Figure 11 and the results, displayed through a heat map, are shown in Figure 12.

## 4. Discussion

Here, we adopted a comprehensive method integrated UPLC-QE-Orbitrap-MS and network pharmacology to reveal the material basis and mechanism of action of the process by which SJWTG treats CG. For the first time, this study has shown that 2,6-bis(4-ethylphenyl)perhydro-1,3,5,7-tetraoxanaphth-4-ylethane-1,2-diol, murrangatin, meranzin hydrate, paeoniflorin, and albiflorin are the key bioactive components of SJWTGs treating CG. Specifically, 2,6-bis(4-ethylphenyl)perhydro-1,3,5,7-tetraoxanaphth-4-ylethane-1,2-diol is a chemical component of an unknown source that is not derived from the 8 herbs included in SJWTGs and has been rarely reported in pharmacological studies. Murrangatin has an antibacterial and anti-inflammatory effect on lipopolysaccharide (LPS)-stimulated inflammation [17], indicating that murrangatin has a potential therapeutic effect on CG with *H. pylori* infection, and might also be a valuable antitumour-promoting agent, inhibiting the proliferation and migration of various cancer cells [18–20]. Meranzin hydrate exhibits high activity against both Gram-positive and Gram-negative bacteria [21] as well as LPS-induced inflammation [22]. According to Medical Subject Headings (https://www.ncbi.nlm.nih.gov/mesh), paeoniflorin is a nonsteroidal anti-inflammatory agent, which can modulate the activation of immune cells and decrease inflammatory medium production via regulating the MAPK/NF-*κ*B pathway, JAK2/STAT3 pathway, and PI3K/Akt/mTOR pathway [23]. Meanwhile, albiflorin could regulate the MAPK/NF-*κ*B signalling pathway by inhibiting the expression of inducible nitric oxide synthase (iNOS), cyclooxygenase-2 (COX-2), TNF-*α*, and IL-6, thereby acting as an anti-inflammatory [24, 25].

We found that the core targets of SJWTGs in treating CG were EGFR, SRC, AKT1, HSP90AA1, MAPK1, and MAPK3. Gastric epithelial EGFR inhibition represents a potential strategy to prevent the development of gastric cancer in *H. pylori*-infected individuals [26]. Human gastric tissues exhibit elevated levels of phosphorylated EGFR during the progression from CG to gastric cancer, which could activate the NF-*κ*B and MAPK1/3 pathways to induce cytokine production and macrophage activation, while EGFR-deficient macrophages display impaired Th1 and Th17 adaptive immune responses to *H. pylori* [27]. SRC plays an important role in the migration of gastric cancer cells by mediating a potential CXCR4-EGFR crosstalk and sequentially activating the EGFR-Akt/ERK axis [28]. Additionally, SRC has been proven to be the kinase mediating the phosphorylation of CagA, a protein delivered into the bacterium-attached gastric epithelial cell by *H. pylori*, in vitro and in vivo [29]. Studies have found that AKT1 gene mutation could drive pathogenicity in gastric cancer [30], and the proliferation and apoptosis of gastric cancer cells could be regulated by affecting the expression level of AKT1 [31, 32]. HSP90AA1, as the target of miR-9-5p, could be targeted to suppress gastric cancer cell proliferation and metastasis [33], and the MAPK1/3 pathway has played crucial roles in *H. pylori* infection, CG, and gastric cancer [26, 27]. Several studies have shown that the tumour growth of gastric cancer could be restrained by inhibiting the expression level of MAPK1 and MAPK3 [34–37]. Moreover, STAT3, HRAS, MAPK14, and MAPK8 are also important in the process of SJWTGs treating CG: pS-STAT3 is essential during the entire pathologic progression from CG to gastric cancer in *H. pylori*-infected mice [38]; HRAS, markedly upregulated in gastric cancer, could enhance the aggressiveness of gastric cancer by activating the VEGFA/PI3K/AKT pathway and Raf-1 signalling [39]; MAPK14 could be a potential biomarker for advanced gastric cancer, as well as a pharmacological target, which could improve the survival rate of patients [40]; and the upregulation of MAPK8 expressed in gastric cancer tissues is relevant to a switch towards a premalignant state in *H. pylori*-infected tissues [41].

Furthermore, we suggest that the MAPK signalling pathway and C-type lectin receptor signalling pathway are vital during the treatment of CG by SJWTGs. The MAPK signalling pathway could be activated by *H. pylori* infection to induce cytokine production and macrophage activation, resulting in the formation of CG [27], which also seems to be a gold target for anticancer therapies, especially for gastric cancer [42]. During the development of *H. pylori*-induced CG into early gastric cancer, Lewis antigens of *H. pylori* LPS could interact with Mincle (Macrophage inducible C-type lectin) and maintain a balance between pro- and anti-inflammatory cytokine production, suggesting that the C-type lectin receptor signalling pathway is closely related to the mechanism employed by *H. pylori* to escape the host innate immune receptors [43]. In addition, the EGFR tyrosine kinase inhibitor resistance, TNF signalling pathway, HIF-1 signalling pathway, Th17 cell differentiation, T-cell receptor signalling pathway, Toll-like receptor signalling pathway, IL-17 signalling pathway, and ErbB signalling pathway have also played crucial roles in the treatment of CG by SJWTGs. The expression of EGFR tyrosine kinase is highly deregulated in gastric cancer tissues, as a result of *H. pylori* [44], indicating that EGFR tyrosine kinase inhibitor resistance could regulate the imbalance of gastric cancer. TNF-*α* can increase the release of pro-inflammatory cytokines, augmenting apoptosis induced by *H. pylori* [45], while monoclonal antibodies targeting TNF-*α* have been investigated for their potential to prevent inflammation-based gastric cancer [46]. As major transcriptional regulators of immunity and inflammation, HIFs are closely related to CG. Studies have found that HIF-1 is protective in *H. pylori*-mediated CG, indicating that the HIF-1 signalling pathway has a potential therapeutical effect [47]. *H. pylori* induces Th17 cell differentiation via infected macrophages, and the number of Th17 cells is positively correlated with the degree of CG [48, 49]. The T-cell receptor signalling pathway could be activated by *H. pylori* protein HP1454, leading to an inflammatory response [50]. Moreover, *H. pylori* infection could initiate chemokine-mediated T lymphocyte trafficking into the inflamed epithelium and induce mucosal injury [51]. Toll-like receptor 4 plays an important role in the Toll-like receptor signalling pathway, which could mediate an inflammatory response in the gastric epithelia induced by *H. pylori* and sequentially contribute to the initiation and progression of gastric cancer cells [52]. As a hallmark cytokine of Th17 cells, IL-17 plays a critical role in the host's defence against bacterial infection [53]. *H. pylori* infection perturbs the balance between T-regulatory and Th17 cells, which causes a spurt of IL-17 and gives rise to CG [54]. Gastric cancer exhibits alterations in the ErbB receptor family and ErbB-related signalling pathways [55]; in particular, the expression level of ErbB2 is increased in gastric cancer, and ErbB2 targeted therapies for gastric cancer have proved highly beneficial [56].

The results of molecular docking showed the strong binding interaction between 5 key bioactive components and 6 core targets, which further verifies the network pharmacology results, to a certain degree. Due to the structural similarity of the 5 key bioactive components, that is, the existence of lactones, hydroxyl groups, and phenyl groups in the structure, they all have good conjugation with the core targets via polar interactions and hydrophobic interactions. Consequently, we suggest that the 5 key bioactive components could be considered as Q-markers. Nevertheless, molecular docking is still based on computer simulation, and the results need to be validated in vivo.

Therefore, we believe that SJWTGs could reduce the inflammatory response of gastric epithelial cells and prevent long-term chronic inflammation to induce cancerization by regulating the MAPK signalling pathway and the C-type lectin receptor signalling pathway as well as their upstream and downstream pathways. This is different to the triple and quadruple therapies that use antibiotics to eradicate *H. pylori*, indicating that SJWTG is also curative in the treatment of CG without *H. pylori* infection. Moreover, we conclude that the pathogenesis of CG may be related to the disorder of the MAPK signalling pathway and the C-type lectin receptor signalling pathway, and that *H. pylori* infection, duodenal-gastric reflux, drugs, and autoimmunity are only the key factors to induce the disorder of these pathways. Furthermore, the preventive effect of SJWTGs on gastric cancer also shows the superiority of preventive treatment of diseases with TCM. Due to the variety of targets, including EGFR, SRC, AKT1, HSP90AA1, MAPK1, and MAPK3, SJWTG is less susceptible to drug resistance during the long-term treatment of CG, suggesting the advantage of Chinese patent medicines. Additionally, 2,6-bis(4-ethylphenyl)perhydro-1,3,5,7-tetraoxanaphth-4-ylethane-1,2-diol, murrangatin, meranzin hydrate, paeoniflorin, and albiflorin have been shown to be the key bioactive components of SJWTGs in treating CG: the results of molecular docking demonstrate their potential therapeutic effects on CG and these components deserved to be further studied and considered as Q-markers, especially the chemical component of an unknown source that is not derived from the 8 herbs, 2,6-bis(4-ethylphenyl)perhydro-1,3,5,7-tetraoxanaphth-4-ylethane-1,2-diol.

## 5. Conclusions

In summary, a comprehensive approach based on UPLC-QE-Orbitrap-MS and network pharmacology has been used to explore the active compounds and potential mechanism of SJWTGs in treating CG. Overall, 95 chemical components were identified by UPLC-QE-Orbitrap-MS, including 6 chemical components of an unknown source that are not derived from the 8 herbs present in SJWTGs. Subsequently, the identified chemical components were further researched by network pharmacology, where it was found that SJWTGs could reduce the inflammatory response of gastric epithelial cells and prevent persistent chronic inflammation to induce cancerization by regulating the MAPK signalling pathway and the C-type lectin receptor signalling pathway, as well as their upstream and downstream pathways, in the treatment of CG, which could also be related to the pathogenesis of CG. The key bioactive components are 2,6-bis(4-ethylphenyl)perhydro-1,3,5,7-tetraoxanaphth-4-ylethane-1,2-diol, a chemical component of an unknown source, murrangatin, meranzin hydrate, paeoniflorin, and albiflorin. The results of molecular docking showed the strong binding interaction between the key bioactive components and the core targets, demonstrating that the key bioactive components deserved to be further studied and considered as Q-markers. Acting on multiple core targets, including EGFR, SRC, AKT1, HSP90AA1, MAPK1, and MAPK3, SJWTG is less susceptible to drug resistance during the long-term treatment of CG, suggesting the advantage of Chinese patent medicines. Furthermore, the preventive effect of SJWTGs on gastric cancer also demonstrates the superiority of the preventive treatment of disease with TCM.

## Figures and Tables

**Table 1:** The identified SJWTG's effective extraction constituents by UPLC-QE-Orbitrap-MS.

No.	*t*_*R*_ (min)	Theoretical m/z<	Measured m/z	Error (ppm)	Ion form	Elemental composition	MS/MS (m/z)	Identification	Source
1	0.89	109.0648	109.0651	2.55	[M+H]^+^	C_7_H_8_O	67.0549, 109.1014, 81.0704, 109.0766, 109.0650	Benzyl alcohol	e

2	1.09	503.1618	503.1619	0.28	[M−H]^−^	C_18_H_32_O_16_	89.0232, 71.0126, 101.0232, 179.0554, 221.0663	D-raffinose	g

3	1.10	665.2146	665.2145	−0.06	[M−H]^−^	C_24_H_42_O_21_	89.0232, 59.0126, 101.0232, 383.1199, 179.0554	Stachyose	g

4	1.11	341.1089	341.1088	−0.31	[M−H]^−^	C_12_H_22_O_11_	89.0232, 71.0126, 101.0233, 179.0553, 341.1096	Sucrose	h

5	1.14	195.0510	195.0502	−4.23	[M−H]^−^	C_6_H_12_O_7_	75.0075, 195.0504, 129.0182, 87.0075, 99.0075	Gluconic acid	g

6	1.16	179.0561	179.0553	−4.36	[M−H]^−^	C_6_H_12_O_6_	59.0126, 71.0126, 89.0232, 179.0553, 101.0232, 119.0340	D-mannose	f/g

7	1.17	191.0561	191.0552	−4.72	[M−H]^−^	C_7_H_12_O_6_	191.0555, 85.0282, 93.0333, 127.0390, 59.0126, 173.0446	Quinic acid	h

8	1.22	152.0567	152.0566	−0.63	[M+H]^+^	C_5_H_5_N_5_O	152.0567, 110.0351, 135.0301, 93.0578, 109.0517, 82.0406	Guanine	f/g

9	1.23	268.1040	268.1037	−1.23	[M+H]^+^	C_10_H_13_N_5_O_4_	268.1036, 137.0454, 136.0617, 85.0288, 73.0290	Adenosine	f/h

10	1.23	353.0725	353.0723	−0.62	[M−H]^−^	C_12_H_18_O_12_	111.0076, 87.0076, 173.0083, 191.0559, 353.1108	2-Hydroxy-2-[2-oxo-2-[(2R, 3R, 4S, 5R)-2,3,4, 5-tetrahydroxy-6-oxohexoxy]ethyl]butanedioic acid	f

11	1.31	169.0142	169.0134	−4.83	[M−H]^−^	C_7_H_6_O_5_	125.0234, 169.01343, 80.9640, 97.0283, 78.9578	Gallic acid	h

12	1.59	137.0597	137.0598	0.32	[M+H]^+^	C_8_H_8_O_2_	137.0458, 81.0704, 95.0859, 91.0547, 119.0353	Phenyl acetic acid	e

13	1.59	407.1195	407.1200	1.30	[M+COOH]^−^	C_15_H_22_O_10_	97.0284, 169.0498, 151.0391, 199.0612, 181.0505,	Catalpol	g

14	1.59	421.1351	421.1358	1.47	[M+COOH]^−^	C_16_H_24_O_10_	165.0549, 345.1193, 89.0231, 195.0695, 123.0442	Desbenzoylalbiflorin	h

15	1.60	116.0706	116.0708	1.77	[M+H]^+^	C_5_H_9_NO_2_	70.0657, 116.0706, 98.9845	Proline	f/g

16	1.61	182.0812	182.0812	0.11	[M+H]^+^	C_9_H_11_NO_3_	136.0756, 123.0441, 165.0544, 147.0439, 182.0806	Tyrosine	f/g

17	1.84	132.1019	132.1019	0.19	[M+H]^+^	C_6_H_13_NO_2_	86.0968, 132.1018, 69.0705, 132.0651	L-norleucine	f/g

18	1.85	191.0197	191.0191	−3.43	[M−H]^−^	C_6_H_8_O_7_	111.0076, 85.0283, 57.0333, 191.0194, 67.0175	Citric acid	h

19	2.13	127.0390	127.0391	0.94	[M+H]^+^	C_6_H_6_O_3_	127.0391, 109.0286, 81.0340, 55.0186, 53.9399	Pyrogallol	h

20	2.35	123.0441	123.0438	−2.16	[M+H]^+^	C_7_H_6_O_2_	123.0552, 123.0805, 95.0495, 105.0451, 53.0393	Benzoic acid	e/h

21	2.68	166.0863	166.0863	0.03	[M+H]^+^	C_9_H_11_NO_2_	120.0809, 103.0645, 93.0703, 166.0857, 79.0547, 121.0646, 149.0600	Phenylalanine	f/g

22	2.80	127.0390	127.0391	1.18	[M+H]^+^	C_6_H_6_O_3_	109.0286, 127.0391, 81.0340, 144.9659, 99.0445	5-hydroxymethyl-2-furaldehyde-13C6	f/g

23	4.64	431.1559	431.1563	0.99	[M−H]^−^	C_19_H_28_O_11_	89.0233, 99.0075, 59.0127, 71.0125, 113.0235	Darendoside A	e

24	4.68	353.0878	353.0881	0.83	[M−H]^−^	C_16_H_18_O_9_	191.0556, 85.0282, 173.0449, 93.0333, 135.0441	Chlorogenic acid	d

25	5.31	342.1700	342.1700	0.07	[M]^+^	C_20_H_24_NO_4_	192.1018, 177.0783, 342.1697, 149.0836, 58.06598	6H-dibenzo(a,g)quinolizinium	c

26	5.78	481.1704	481.1707	0.59	[M+H]^+^	C_23_H_28_O_11_	105.0338, 133.0648, 151.0754, 197.0808, 179.0701	Albiflorin	h

27	5.81	479.1559	479.1561	0.45	[M−H]^−^	C_23_H_28_O_11_	121.0285, 93.0334	Mudanpioside I	h

28	5.94	342.1700	342.1701	0.25	[M]^+^	C_20_H_24_NO_4_	265.0859, 297.1124, 237.0911, 342.1711, 282.0883	Magnoflorine	c

29	6.12	579.1708	579.1707	−0.31	[M+H]^+^	C_27_H_30_O_14_	441.1092, 363.0866, 321.0771, 309.0754, 279.0652	5,7-Dihydroxy-2-phenyl-6,8-bis[3 4, 5-trihydroxy-6-(hydroxymethyl)oxan-2-yl] chromen-4-one	—

30	6.24	163.0401	163.0393	−4.83	[M−H]^−^	C_9_H_8_O_3_	119.0493, 163.0394, 93.0335, 116.9274, 63.3064	p-Coumaric acid	a/h

30	6.26	165.0546	165.0547	0.66	[M+H]^+^	C_9_H_8_O_3_	147.0441, 119.0493, 95.0497, 91.0547, 165.0914	p-Coumaric acid	a/h

31	6.31	356.1492	356.1494	0.28	[M+H]^+^	C_20_H_21_NO_5_	356.1481, 338.1399, 275.0698, 206.0808, 188.0707	5,7,8,15-Tetrahydro-4-hydroxy-3-methoxy-6-methyl [1, 3] benzodioxolo[5, 6-e][2]benzazecin-14(6H)-one	c

32	6.32	431.0984	431.0990	1.39	[M−H]^−^	C_21_H_20_O_10_	283.0617, 117.0338, 161.0242, 121.0288, 135.0440	Vitexin	c

33	6.39	547.1457	547.1466	1.53	[M−H]^−^	C_26_H_28_O_13_	337.0724, 281.0826, 367.0831, 457.1163, 487.1229	Chrysin-6-C-arabinoside-8-C-glucoside	e

34	6.49	461.0725	461.0734	1.76	[M−H]^−^	C_21_H_18_O_12_	285.0410, 113.0233, 239.0358, 267.0302, 284.0313	Scutellarin	e

34	6.49	463.0871	463.0872	0.12	[M+H]^+^	C_21_H_18_O_12_	287.0548, 123.0078, 119.0493, 169.0137, 141.0185, 91.0547	Scutellarin	e

35	6.51	623.1981	623.1966	−2.49	[M−H]^−^	C_29_H_36_O_15_	161.0238, 133.02866, 135.0443, 461.1667, 179.0343	Acteoside	e/g

36	6.60	260.1281	260.1282	0.42	[M+H]^+^	C_15_H_17_NO_3_	188.0706, 242.1175, 260.1278, 134.0604, 176.0706	Ribalinine	c

37	6.60	183.0652	183.0653	0.57	[M+H]^+^	C_9_H_10_O_4_	95.0496, 123.0442, 140.0468, 155.0704, 183.0637	Syringaldehyde	e

38	6.64	161.0244	161.0236	−4.95	[M−H]^−^	C_9_H_6_O_3_	161.0238, 133.0286, 123.0442, 117.0336, 162.0188, 89.0384, 95.0491	Umbelliferone	a

39	6.96	356.1856	356.1857	0.27	[M]^+^	C_21_H_26_NO_4_	265.0857, 311.1277, 296.1040, 281.0806, 237.0909, 207.0803	4H-dibenzo(de,g)quinolinium	c

40	7.00	579.1708	579.1714	0.96	[M+H]^+^	C_27_H_30_O_14_	267.0651, 297.0757, 321.0761, 363.0856, 345.0749	Puerarin 4′-O-glucoside	—

41	7.03	547.1457	547.1466	1.53	[M−H]^−^	C_26_H_28_O_13_	281.0825, 337.0733, 427.1056, 309.0793, 367.0857	Chrysin-6-C-glucoside-8-C-arabinoside	e

42	7.10	493.1341	493.1342	0.22	[M+H]^+^	C_23_H_24_O_12_	331.0812, 316.0574, 270.0519, 298.0473, 287.0544	Viscidulin II	e

43	7.24	609.1825	609.1838	2.11	[M−H]^−^	C_28_H_34_O_15_	301.0723, 151.0029, 242.0586, 286.0483, 257.0827, 325.0732	Hesperidine	c

44	7.36	477.1028	477.1028	0.18	[M+H]^+^	C_22_H_20_O_12_	301.0703, 286.0471, 186.0163, 168.0051, 121.0284	2′,5-Dihydroxy-6-methoxy-7-(beta-D-glucurono pyranosyloxy)flavone	e

45	7.45	301.0707	301.0708	0.55	[M+H]^+^	C_16_H_12_O_6_	286.0473, 301.0712, 186.0165, 168.0056, 105.0342	5,7,2′-trihydroxy-6-methoxyflavone	e

46	7.52	187.0976	187.0970	−3.22	[M−H]^−^	C_9_H_16_O_4_	125.0963, 187.0970, 97.0648, 187.8727, 126.0994	Azelaic acid	f/g

47	7.55	481.1704	481.1706	0.38	[M+H]^+^	C_23_H_28_O_11_	105.0339, 133.0648, 151.0755, 197.0812, 161.0598	Paeoniflorin	h

48	7.60	370.1649	370.1650	0.38	[M+H]^+^	C_21_H_23_NO_5_	188.0705, 370.1647, 189.0787, 206.0810, 352.1542	Allocryptopine	c

49	7.65	287.0550	287.0550	0.02	[M+H]^+^	C_15_H_10_O_6_	287.0548, 153.0182, 241.0496, 135.0439, 269.0451	Luteolin	e/h

50	7.66	259.0965	259.0964	−0.29	[M−H_2_O+H]^+^	C_15_H_16_O_5_	189.0546, 131.0492, 103.0546, 105.0703, 128.0620	Murrangatin	b

51	7.86	447.0922	447.0920	−0.53	[M+H]^+^	C_21_H_18_O_11_	271.0600, 123.0077, 141.0183, 253.0481, 169.0133	Baicalin	e

52	7.89	334.1074	334.1076	0.76	[M+H]^+^	C_20_H_15_NO_4_	334.10739, 291.0888, 319.0836, 276.0657, 262.0859, 304.0626	Norchelerythrine	c

53	7.97	431.0984	431.0987	0.74	[M−H]^−^	C_21_H_20_O_10_	269.0460, 169.0653, 223.0402, 241.0505, 251.0352	Oroxin A	e

54	7.98	271.0601	271.0600	−0.26	[M+H]^+^	C_15_H_10_O_5_	271.0599, 123.0078, 169.0125, 253.0492, 225.0546	Baicalein	e

55	8.00	347.0761	347.0754	−2.29	[M+H]^+^	C_17_H_14_O_8_	347.0758, 314.0413, 289.0346, 286.0472, 233.0440	Viscidulin III	e

56	8.02	261.1121	261.1122	0.29	[M−H_2_O+H]^+^	C_15_H_18_O_5_	189.0546, 131.0493, 103.0546, 159.0442, 243.1017	Meranzin hydrate	b

57	8.20	332.0917	332.0918	0.20	[M]^+^	C_20_H_14_NO_4_	332.0918, 274.0866, 317.0683, 304.0973, 291.1183, 216.0808, 246.0914, 261.0790	Sanguinarine	c

58	8.30	273.0758	273.0758	0.33	[M+H]^+^	C_15_H_12_O_5_	273.0756, 169.0131, 103.0546, 123.0078, 141.0184	Dihydrobaicalein	e

59	8.37	285.0758	285.0759	0.42	[M+H]^+^	C_16_H_12_O_5_	270.0523, 285.0756, 179.0491, 242.0574, 168.0568, 140.0620	Wogonin	e

60	8.40	247.0965	247.0963	−0.63	[M+H]^+^	C_14_H_14_O_4_	247.0963, 175.0390, 229.0858, 176.0467, 147.0435	Marmesin	a

61	8.50	348.1230	348.1227	−1.02	[M]^+^	C_21_H_18_NO_4_	348.1227, 332.0913, 304.0964, 290.0811, 318.0760, 274.0860	Nitidine	c

62	8.55	345.0969	345.0971	0.67	[M+H]^+^	C_18_H_16_O_7_	330.0733, 345.0966, 259.0601, 287.0550, 315.0497	Pachypodol	a

63	8.58	461.1078	461.1080	0.35	[M+H]^+^	C_22_H_20_O_11_	270.0521, 285.0755, 186.0158, 168.0053, 242.0572	Oroxylin A-7-o-beta-D-glucuronide	e

64	8.58	246.0761	246.0762	0.31	[M+H]^+^	C_13_H_11_NO_4_	231.0526, 246.0760, 216.0290, 188.0339, 120.0811	Haplopine	c

65	8.59	447.1286	447.1289	0.82	[M+H]^+^	C_22_H_22_O_10_	270.0521, 285.0756, 186.0163, 168.0052, 242.0574	5-hydroxy-6-methoxy-2-phenyl-7-[3, 4, 5-trihydroxy-6-(hydroxymethyl)oxan-2-yl]oxychromen-4-one	e

66	8.64	431.0973	431.0978	1.11	[M+H]^+^	C_21_H_18_O_10_	255.0650, 153.0182, 103.0546, 129.0038, 187.0754	Chrysin 7-glucuronide	e

67	8.66	253.0506	253.0509	1.02	[M−H]^−^	C_15_H_10_O_4_	253.0509, 63.0228, 143.0491, 107.0129, 119.0492, 209.0611	Chrysin	e/h

68	8.77	477.1028	477.1030	0.58	[M+H]^+^	C_22_H_20_O_12_	301.0703, 286.0469, 202.0107, 85.0289, 184.0002	5,7,8-Trihydroxy-6-methoxyflavone-7-O-glucuronopyranoside	e

69	8.93	389.1231	389.1233	0.43	[M+H]^+^	C_20_H_20_O_8_	389.1231, 373.0901, 359.0741, 343.0790, 341.0682	Demethylnobiletin	b

70	9.01	491.1184	491.1188	0.75	[M+H]^+^	C_23_H_22_O_12_	315.0860, 285.0391, 282.0521, 300.0629, 257.0440	5,7-Dihydroxy-6,8-dimethoxyflavone-7-O-glucuronopyranoside	e

71	9.03	315.0863	315.0861	−0.71	[M+H]^+^	C_17_H_14_O_6_	315.0864, 285.0390, 257.0456, 197.0599, 182.9934	5,8-Dihydroxy-6,7-dimethoxyflavone	e

72	9.15	259.0965	259.0962	−1.26	[M+H]^+^	C_15_H_14_O_4_	131.0492, 189.0545, 103.0546, 231.1014, 105.0701	Murralongin	b

73	9.35	299.0561	299.0565	1.27	[M−H]^−^	C_16_H_12_O_6_	284.0329, 136.9872, 65.0021, 299.0574, 212.0481	Hispidulin	e

74	9.53	113.0346	113.0347	1.56	[M+H]^+^	C_4_H_4_N_2_O_2_	113.0348, 70.0294, 96.0086, 95.0494, 112.5189	Uracil	c

75	9.83	233.1536	233.1534	−0.76	[M+H]^+^	C_15_H_20_O_2_	187.1480, 131.0856, 233.1534, 215.1428, 197.1326, 205.1587	Costunolide	d

76	9.98	299.0561	299.0564	0.97	[M−H]^−^	C_16_H_12_O_6_	284.0329, 171.0444, 299.0555, 239.0363, 256.0374	Hydroxygenkwanin	h

77	10.04	230.0812	230.0812	0.22	[M+H]^+^	C_13_H_11_NO_3_	230.0810, 215.0575, 200.0341, 186.0549, 172.0391	*γ*-Fagarine	c

78	10.48	336.1442	336.1436	−1.71	[M+NH_4_]^+^	C_17_H_18_O_6_	189.0544, 131.0492, 231.1041, 203.0701, 336.1852	Hainanmurpanin	b

79	10.53	271.0601	271.0599	−0.59	[M+H]^+^	C_15_H_10_O_5_	271.0598, 123.0079, 253.0500, 169.0130, 272.0642	Norwogonin	e

80	10.56	403.1387	403.1390	0.51	[M+H]^+^	C_21_H_22_O_8_	403.1383, 373.0915, 327.0856, 388.1146, 342.1088	Nobiletin	b

81	10.63	329.0667	329.0671	1.32	[M−H]^−^	C_17_H_14_O_7_	314.0434, 271.0252, 299.0200, 243.0292, 199.0398	Aurantio-obtusin	h

82	10.73	200.0706	200.0706	−0.18	[M+H]^+^	C_12_H_9_NO_2_	200.0704, 185.0471, 128.9508, 161.0470, 144.0806	Dictamine	c

83	11.30	375.1074	375.1075	0.02	[M+H]^+^	C_19_H_18_O_8_	375.1069, 314.0779, 360.0837, 298.0474, 345.0599	5,7-Dihydroxy-6,8,2′, 3′-tetramethoxyflavone	e

84	11.45	285.0758	285.0754	−1.19	[M+H]^+^	C_16_H_12_O_5_	270.0519, 285.0754, 168.0866, 242.0565, 186.0158	Oroxylin A	e

85	11.55	253.0506	253.0508	0.58	[M−H]^−^	C_15_H_10_O_4_	253.0508, 63.0228, 209.1540, 143.0495, 107.0123	7,8-Dihydroxyflavone	e

86	11.62	274.2741	274.2736	−1.70	[M+NH_4_]^+^	C_16_H_32_O_2_	274.2736, 88.0761, 70.0658, 57.0706, 106.0865, 256.2632	Palmitic acid	a/b/c/h

87	12.79	231.1016	231.1016	−0.05	[M+H]^+^	C_14_H_14_O_3_	189.0544, 131.0491, 103.0545, 231.1015, 203.0701	7-Methoxy-4-methyl-8-prop-2-enylchromen-2-one	—

88	13.16	415.2115	415.2113	−0.59	[M+H]^+^	C_24_H_30_O_6_	119.0856, 91.0546, 117.0706, 133.0649, 104.0624, 103.0544	2,6-bis(4-ethylphenyl)perhydro-1,3,5,7-tetraoxanaphth-4-ylethane-1,2-diol	—

89	14.21	231.1380	231.1379	−0.29	[M+H]^+^	C_15_H_18_O_2_	185.1323, 231.1350, 157.1010, 195.1164, 165.0696	Dehydrocostus lactone	d

90	15.37	350.1387	350.1385	−0.56	[M+H]^+^	C_21_H_19_NO_4_	334.1069, 335.1144, 318.0755, 319.1194, 318.1122	Dihydrochelerythrine	c

91	16.81	279.1591	279.1578	−4.78	[M+H]^+^	C_16_H_22_O_4_	149.0233, 279.1203, 121.1013, 151.0752, 205.1561, 150.3822	Dibutyl phthalate	a

92	17.00	199.0612	199.0607	−2.60	[M−H]^−^	C_9_H_12_O_5_	155.1069, 162.8378, 199.8049, 137.0963, 108.8987	Rehmaglutin C	g

93	17.61	281.2475	281.2473	−0.81	[M+H]^+^	C_18_H_32_O_2_	281.1357, 97.0652, 245.2259, 263.2367, 264.2405	Linoleic acid	a

94	18.80	282.2791	282.2790	−0.54	[M+H]^+^	C_18_H_35_NO	69.0706, 55.0551, 83.0861, 57.0707, 97.1016, 282.2788, 247.2418, 265.2527	Oleamide	—

95	18.84	123.1168	123.1169	0.43	[M+H]^+^	C_9_H_14_	123.0807, 81.0705, 95.0860, 67.0550, 121.0651, 108.0573	1,2,3,4-Tetramethylcyclopenta-1,3-diene	—

a: *Melicope pteleifolia*; b: *Murraya exotica*; c: *Zanthoxylum nitidum*; d: *Dolomiaea costus*; e: *Scutellaria baicalensis*; f: *Poria cocos*; g: *Rehmannia glutinosa*; h: *Paeonia lactiflora*.

**Table 2:** Molecular docking results of 5 key bioactive compounds of SJWTG in treating CG.

Compounds	EGFR (1M17)	SRC (1YOL)	AKT1 (3QKK)	HSP90AA1 (1UYM)	MAPK1 (6SLG)	MAPK3 (4QTB)
2,6-Bis(4-ethylphenyl)perhydro-1,3,5,7-tetraoxanaphth-4-ylethane-1,2-diol	−4.698	−5.827	−4.935	−6.445	−5.671	−6.157
Murrangatin	−7.037	−6.351	−5.387	−7.806	−5.982	−5.944
Meranzin hydrate	−6.693	−6.195	−5.849	−7.800	−6.602	−6.212
Paeoniflorin	−6.278	−5.124	−5.356	−7.876	−7.262	−5.331
Albiflorin	−5.839	−5.300	−5.580	−7.383	−6.599	−6.200

## Data Availability

The data generated or analysed during this study are included within the article and its supplementary information files.

## Abbreviations

SJWTGs: San-Jiu-Wei-Tai granules
CG: Chronic gastritis
*H. pylori*: *Helicobacter pylori*
SPEM: Spasmolytic polypeptide-expressing metaplasia
Q-marker: Quality marker
TCM: Traditional Chinese medicine
Co., Ltd: Company limited
UPLC-QE-Orbitrap-MS: Ultra-high performance liquid chromatography-Q exactive-mass spectrometry
ESI: Electrospray ionization
MS: Mass spectrometry
MS2: The secondary mass spectrometry
FWHM: Full width at half maximum
PPI: Protein-protein interaction
DC: Degree centrality
BC: Betweenness centrality
CC: Closeness centrality
GO: Gene ontology
KEGG: Kyoto Encyclopedia of Genes and Genomes
EGFR: Epidermal growth factor receptor
SRC: Proto-oncogene tyrosine-protein kinase Src
AKT1: Protein kinase B
HSP90AA1: Heat shock protein 90-alpha
MAPK1: Mitogen-activated protein kinase 1
MAPK3: Mitogen-activated protein kinase 3
2D: Two-dimensional
3D: Three-dimensional
BPI: Base peak intensity chromatogram
RDA: Retro-Diels–Alder
STAT3: Signal transducer and activator of transcription 3
BP: Biological process
MF: Molecular function
CC: Cellular components
MAPK14: Mitogen-activated protein kinase 14
MAPK8: Mitogen-activated protein kinase 8
TNF: Tumour necrosis factor
VEGFA: Vascular endothelial growth factor A
HRAS: GTPase HRas
JUN: Transcription factor Jun
IL6: Interleukin-6
MAPK: Mitogen-activated protein kinase
LPS: Lipopolysaccharide
MAPK/NF-*κ*B: Mitogen-activated protein kinase/nuclear factor-*κ*B
JAK2/STAT3: Tyrosine-protein kinase JAK2/signal transducer and activator of transcription 3
PI3K/Akt/mTOR: Phosphatidylinositol 3 kinase/protein kinase B/mammalian target of rapamycin
iNOS: Inducible nitric oxide synthase
COX-2: Cyclooxygenase-2
TNF-*α*: Tumour necrosis factor-*α*
MAPK1/3: Mitogen-activated protein kinase 1/3
Th1: T helper cell 1
Th17: T helper cell 17
CXCR4-EGFR: C-X-C chemokine receptor type 4-epidermal growth factor receptor
EGFR-Akt/ERK: Epidermal growth factor receptor-protein kinase B/extracellular regulated protein kinase
CagA: Cytotoxin-associated gene A
VEGFA/PI3K/AKT: Vascular endothelial growth factor A/phosphatidylinositol 3 kinase/protein kinase B
Raf-1: RAF proto-oncogene serine/threonine-protein kinase
HIF-1: Hypoxia-inducible factor 1
ErBb: Epidermal growth factor receptor
ErBb2: Receptor tyrosine-protein kinase erbB-2
IL-17: Interleukin-17.

## Supplementary Materials

Supplementary Materials Figure S1: The chemical structures of 95 identified compounds. Figure S2: The MS/MS spectrum of marmesin in positive ion mode and probable fragmentation pathway. Figure S3: The MS/MS spectrum of murralongin in positive ion mode and probable fragmentation pathway. Figure S4: The MS/MS spectrum of nitidine in positive ion mode and probable fragmentation pathway. Figure S5: The MS/MS spectrum of costunolide in positive ion mode and probable fragmentation pathway. Figure S6: The MS/MS spectrum of scutellarin in positive ion mode and probable fragmentation pathway. Figure S7: The MS/MS spectrum of guanine in positive ion mode and probable fragmentation pathway. Figure S8: The MS/MS spectrum of acteoside in negative ion mode and probable fragmentation pathway. Figure S9: The MS/MS spectrum of albiflorin in positive ion mode and probable fragmentation pathway. Figure S10: The MS/MS spectrum of 2,6-bis(4-ethylphenyl)perhydro-1,3,5,7-tetraoxanaphth-4-ylethane-1,2-diol in positive ion mode and probable fragmentation pathway. Table S1: Abbreviated names of 95 identified compounds. Table S2: Information of the core targets.
