# Supplementary material for: Investigating the Active Substance and Mechanism of San-Jiu-Wei-Tai Granules via UPLC-QE-Orbitrap-MS and Network Pharmacology
Source: Evid Based Complement Alternat Med. 2022 Oct 17;2022:1487903. doi: 10.1155/2022/1487903 (PMC9592199; doi:10.1155/2022/1487903)
Supplement: Supplementary Materials — Figure S1: The chemical structures of 95 identified compounds. Figure S2: The MS/MS spectrum of marmesin in positive ion mode and probable fragmentation pathway. Figure S3: The MS/MS spectrum of murralongin in positive ion mode and probable fragmentation pathway. Figure S4: The MS/MS spectrum of nitidine in positive ion mode and probable fragmentation pathway. Figure S5: The MS/MS spectrum of costunolide in positive ion mode and probable fragmentation pathway. Figure S6: The MS/MS spectrum of scutellarin in positive ion mode and probable fragmentation pathway. Figure S7: The MS/MS spectrum of guanine in positive ion mode and probable fragmentation pathway. Figure S8: The MS/MS spectrum of acteoside in negative ion mode and probable fragmentation pathway. Figure S9: The MS/MS spectrum of albiflorin in positive ion mode and probable fragmentation pathway. Figure S10: The MS/MS spectrum of 2,6-bis(4-ethylphenyl)perhydro-1,3,5,7-tetraoxanaphth-4-ylethane-1,2-diol in positive ion mode and probable fragmentation pathway. Table S1: Abbreviated names of 95 identified compounds. Table S2: Information of the core targets. [file 1487903.f1.doc]

# Supplementary Materials

# Investigating the active substance and mechanism of San-Jiu-Wei-Tai granules via UPLC-QE-Orbitrap-MS and network pharmacology

Gengyuan Yu,^1^ Tonghua Zhang,^1^ Haoran Xu,^1^ Yuelin Bi,^1^ Xin Feng,^1^ Jiaqi Wang,^1^ Tianyi Li,^1^ Chenning Zhang,^1,2*^ and Yikun Sun^1*^

^1^ School of Chinese Materia Medica, Beijing University of Chinese Medicine, Beijing, China.
^2^ Department of Pharmacy, Xiangyang No. 1 People's Hospital, Hubei University of Medicine, Xiangyang, China.

Correspondence should be addressed to Yikun Sun; Sunyk@bucm.edu.cn and Chenning Zhang; zhangcn1118@163.com





Figure S1: The chemical structures of 95 identified compounds.


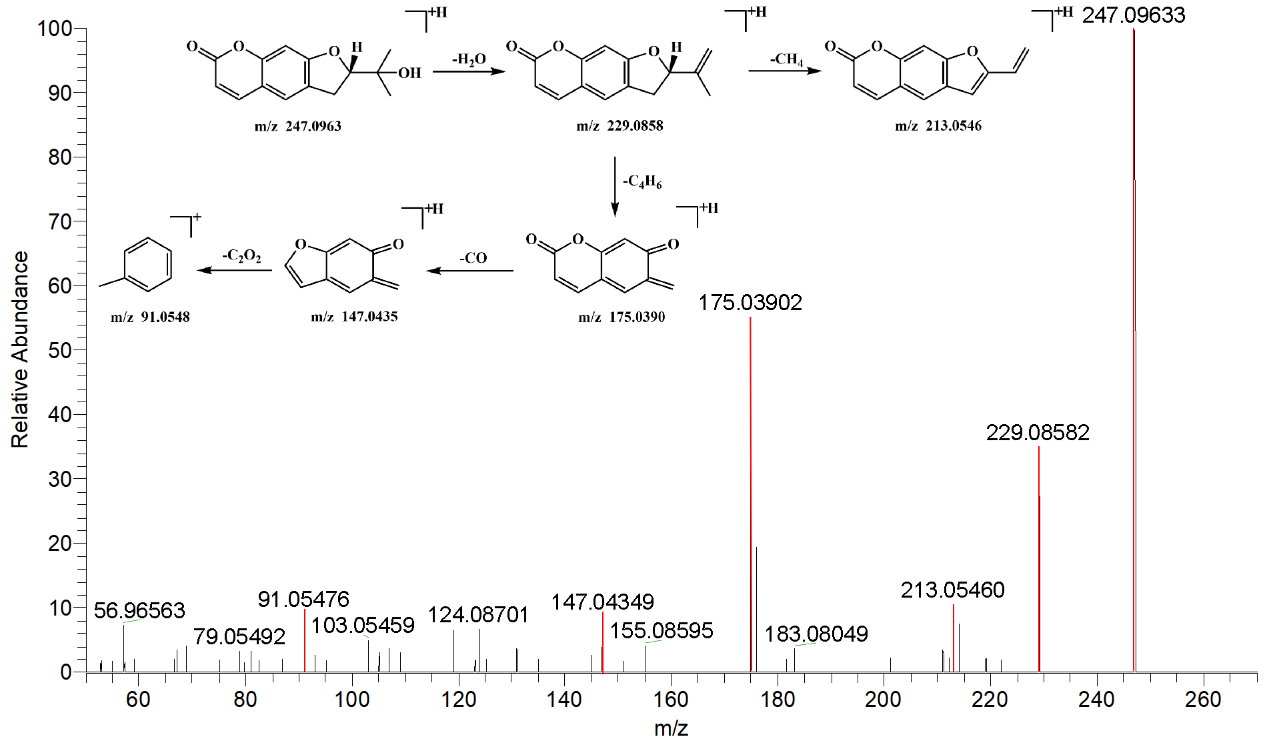


Figure S2: The MS/MS spectrum of marmesin in positive ion mode and probable fragmentation pathway.


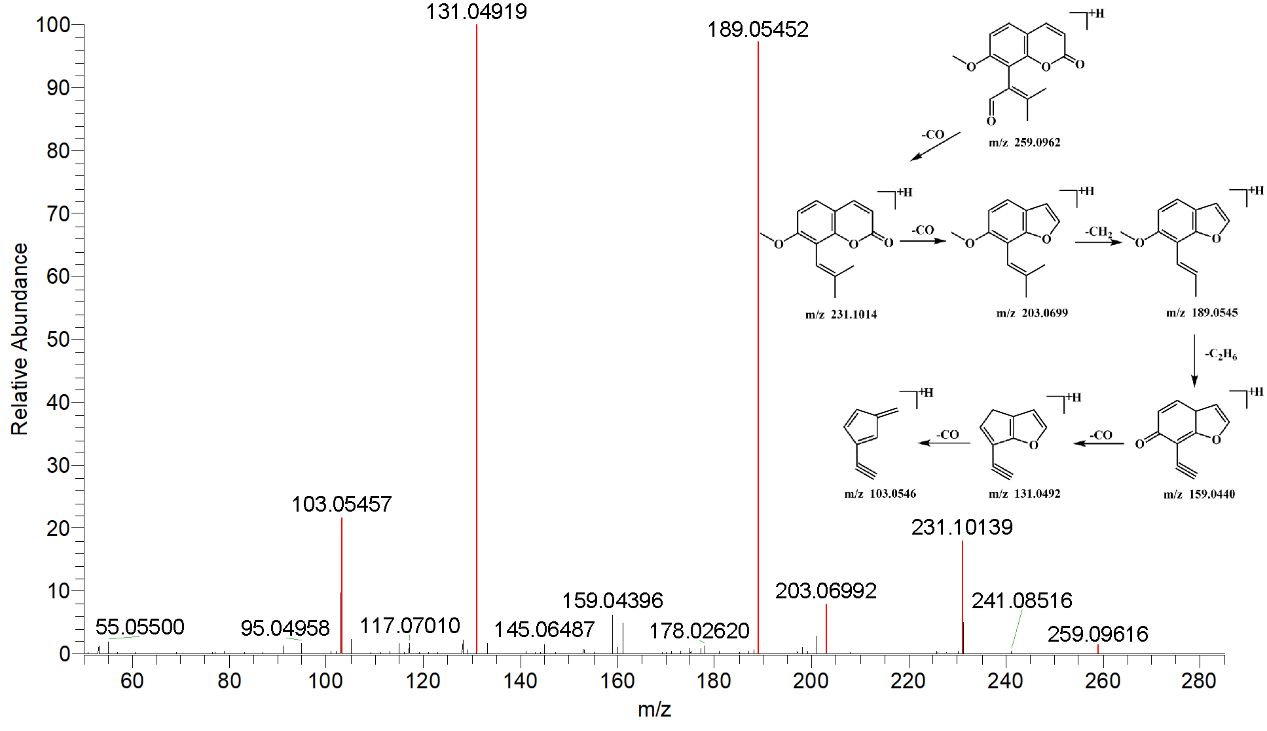


Figure S3: The MS/MS spectrum of murralongin in positive ion mode and probable fragmentation pathway.


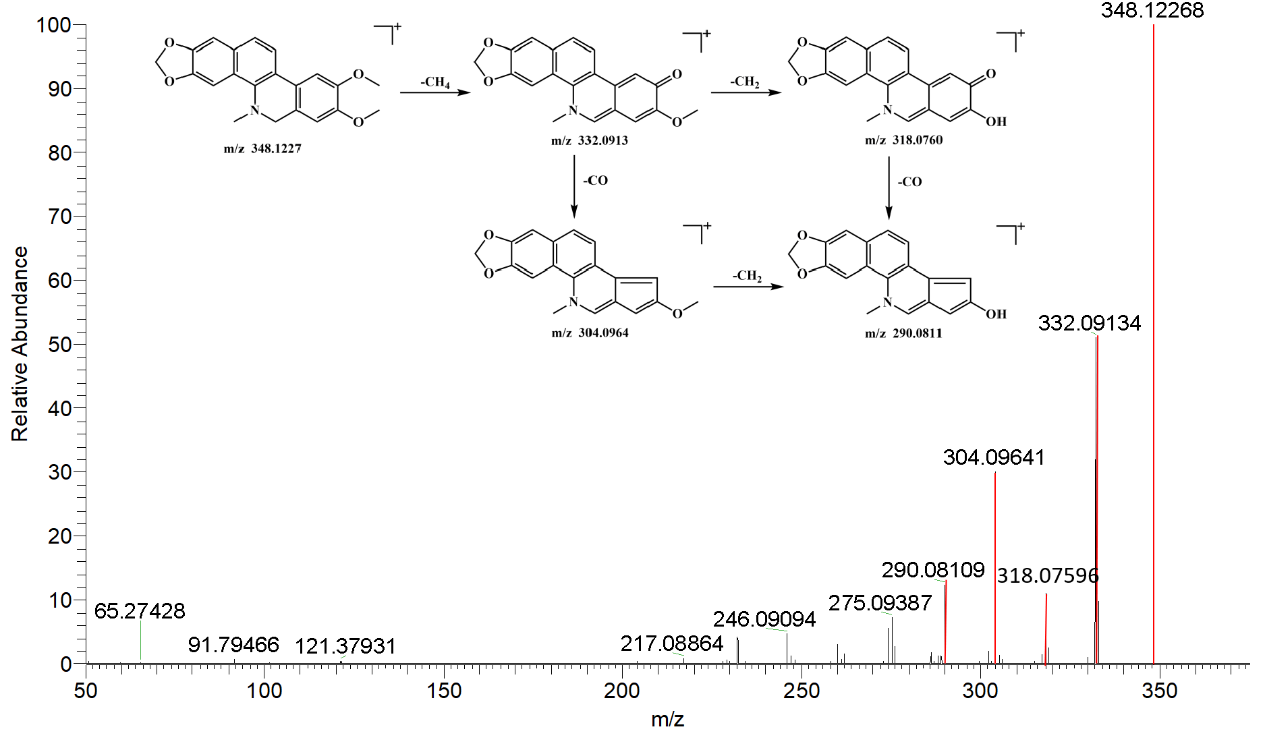


Figure S4: The MS/MS spectrum of nitidine in positive ion mode and probable fragmentation pathway.


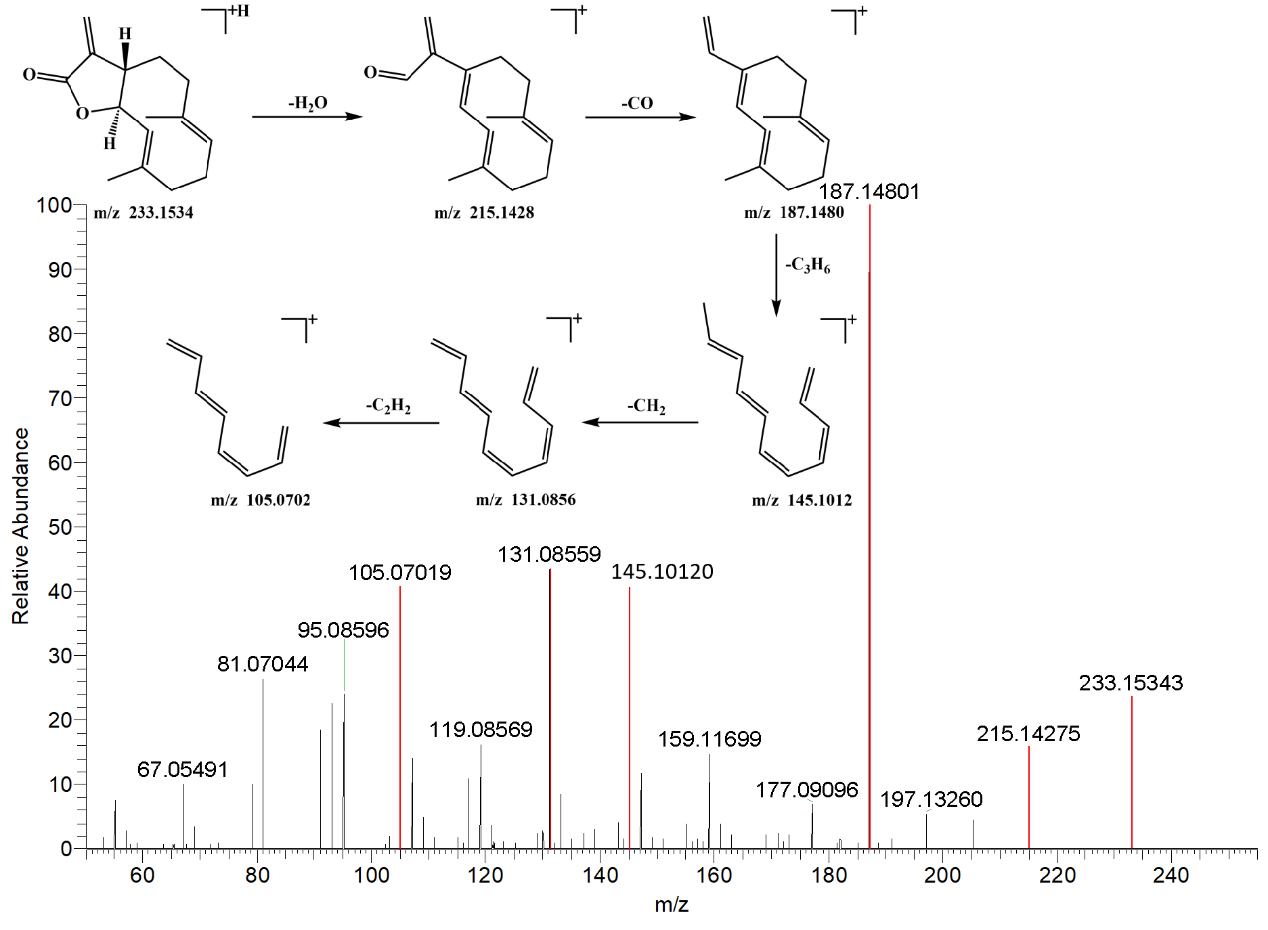


Figure S5: The MS/MS spectrum of costunolide in positive ion mode and probable fragmentation pathway.


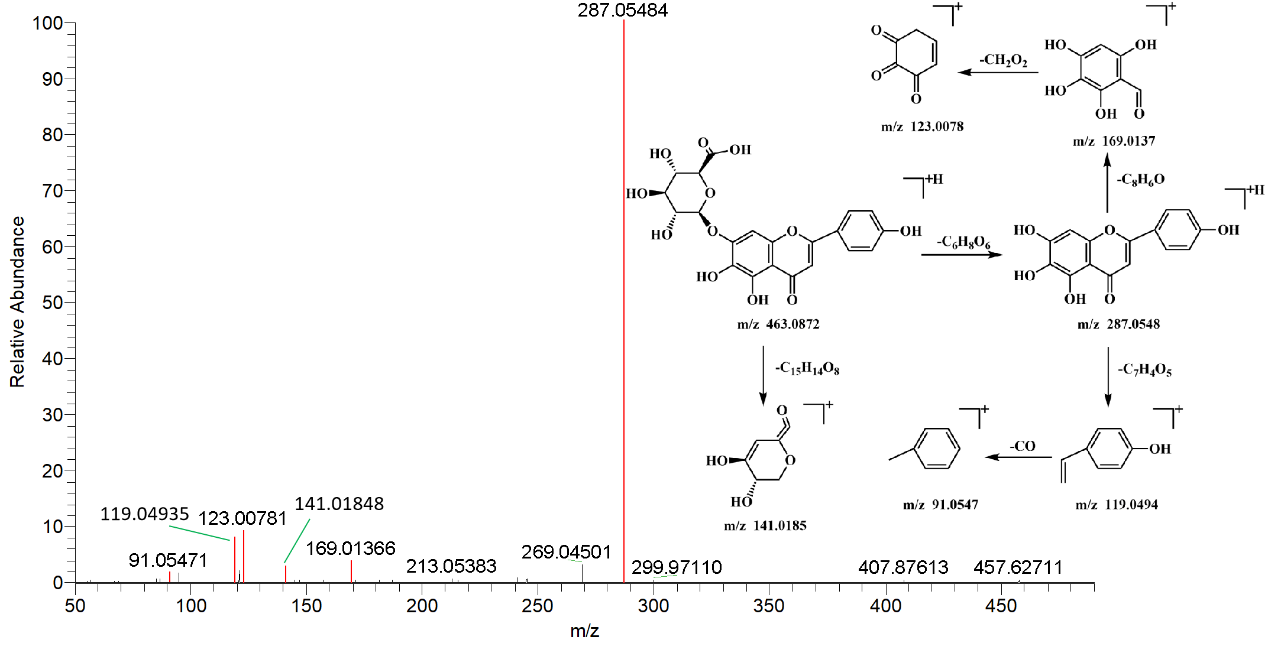


Figure S6: The MS/MS spectrum of scutellarin in positive ion mode and probable fragmentation pathway.


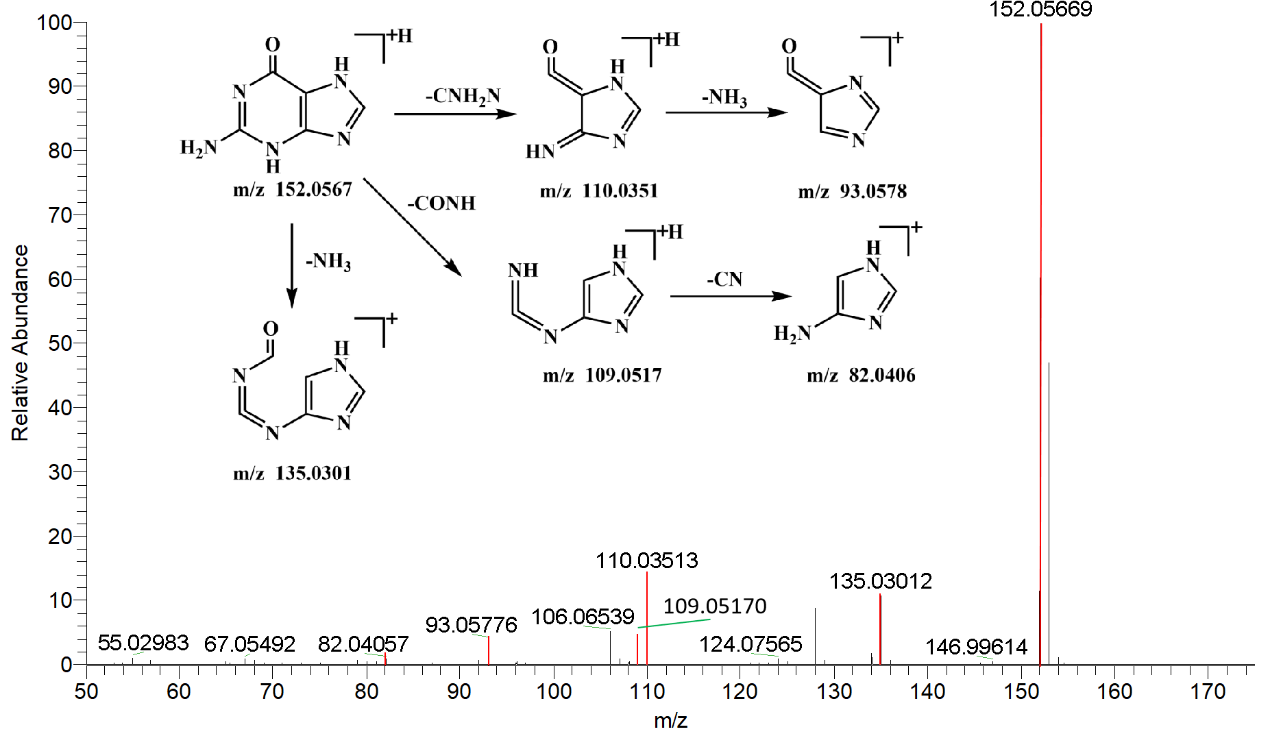


Figure S7: The MS/MS spectrum of guanine in positive ion mode and probable fragmentation pathway.


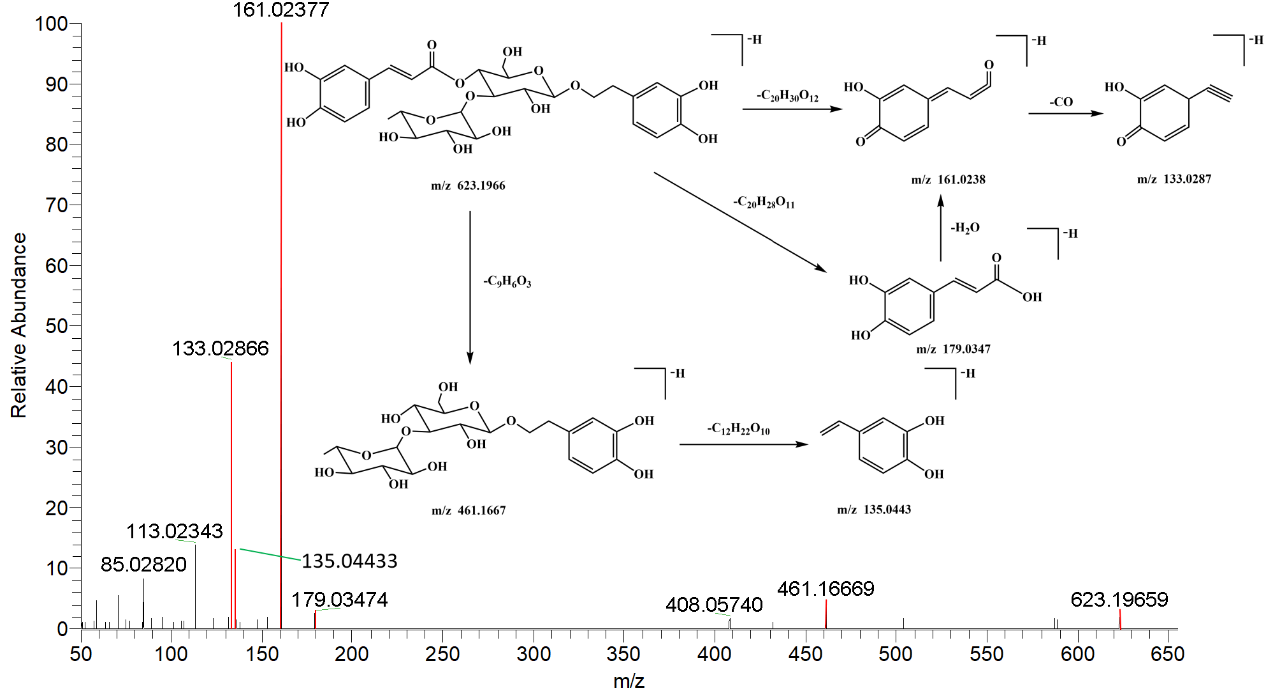


Figure S8: The MS/MS spectrum of guanine in positive ion mode and probable fragmentation pathway.


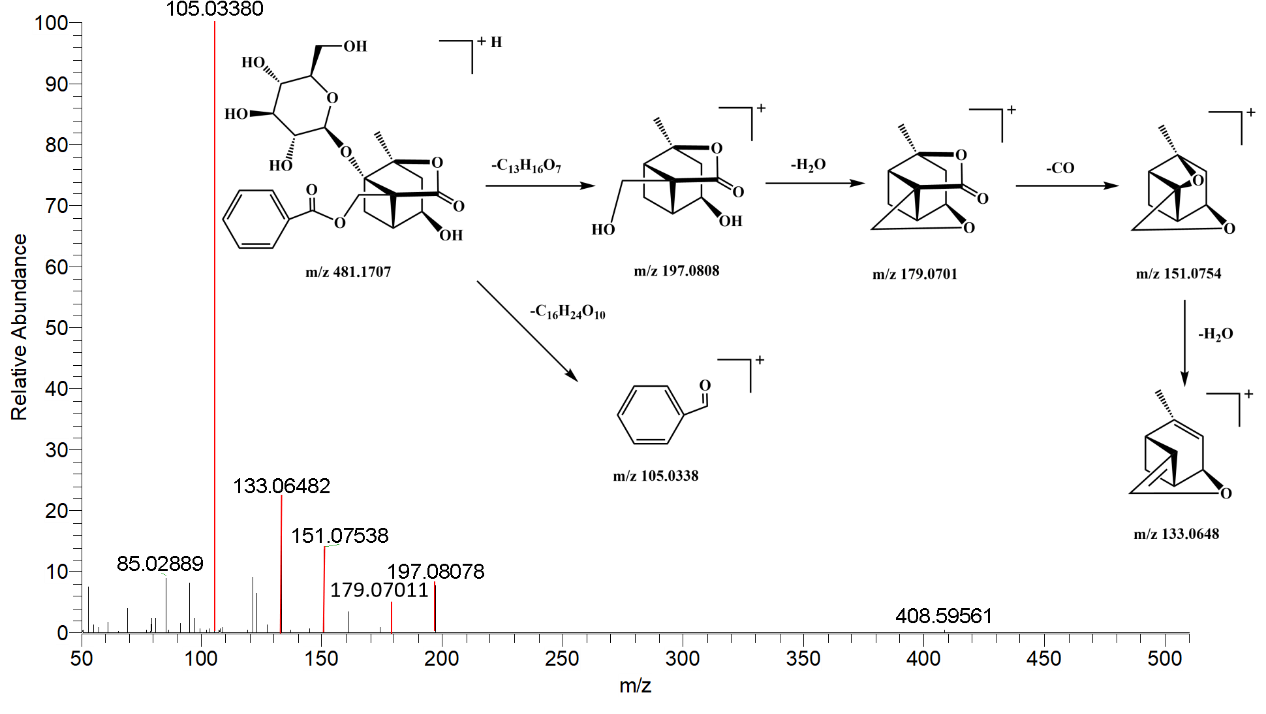


Figure S9: The MS/MS spectrum of albiflorin in positive ion mode and probable fragmentation pathway.


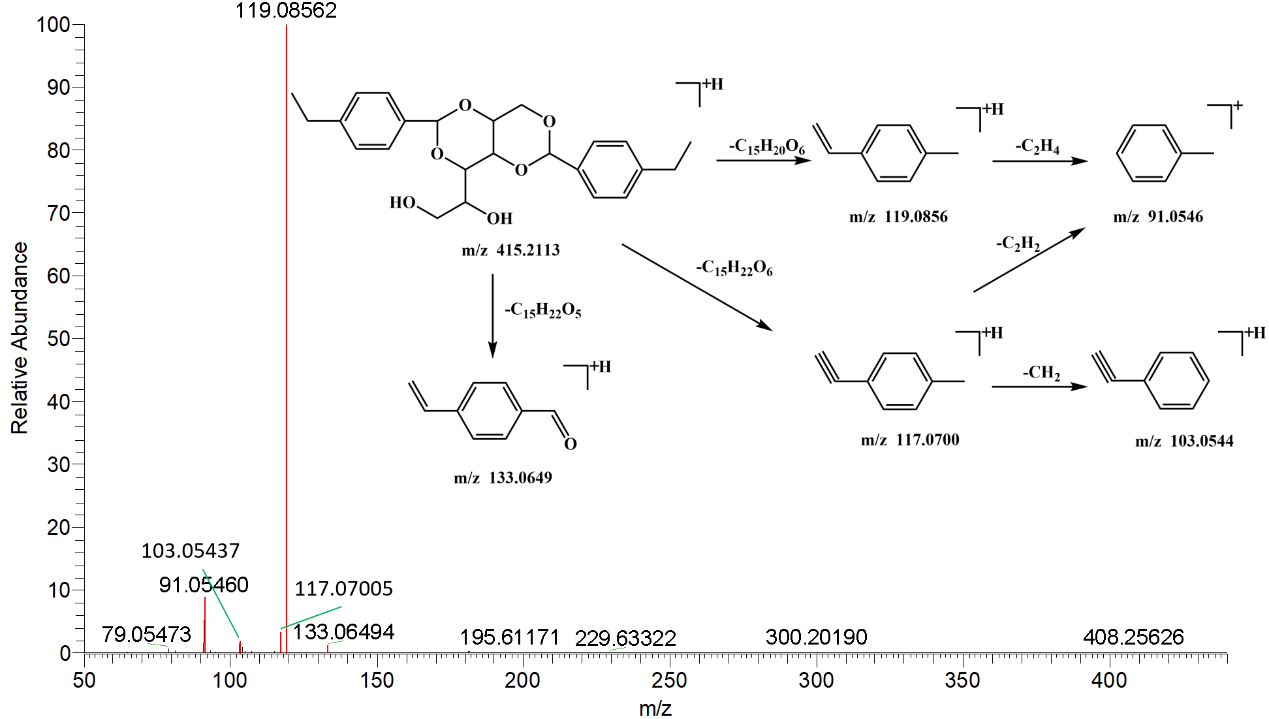


Figure S10: The MS/MS spectrum of 2,6-Bis(4-ethylphenyl)perhydro-1,3,5,7-tetraoxanaphth-4-ylethane-1,2-diol in positive ion mode and probable fragmentation pathway.

*Table S1: Abbreviated names of 95 identified compounds.*

| Compound | Abbreviated name | Compound | Abbreviated name |
| --- | --- | --- | --- |
| luteolin | A1 | syringaldehyde | HQ5 |
| chrysin | A2 | viscidulin II | HQ6 |
| benzoic acid | A3 | 2',5-dihydroxy-6-methoxy-7-(beta-D-glucurono pyranosyloxy)flavone | HQ7 |
| p-coumaric acid | B1 | baicalin | HQ8 |
| sucrose | BS1 | oroxin A | HQ9 |
| hydroxygenkwanin | BS10 | D-mannose | I1 |
| aurantio-Obtusin | BS11 | guanine | I2 |
| quinic acid | BS2 | proline | I3 |
| gallic acid | BS3 | tyrosine | I4 |
| desbenzoylalbiflorin | BS4 | L-norleucine | I5 |
| citric acid | BS5 | phenylalanine | I6 |
| pyrogallol | BS6 | 5-hydroxymethyl-2-furaldehyde-13C6 | I7 |
| albiflorin | BS7 | azelaic acid | I8 |
| mudanpioside I | BS8 | murralongin | JLX1 |
| paeoniflorin | BS9 | hainanmurpanin | JLX2 |
| adenosine | C1 | nobiletin | JLX3 |
| palmitic acid | D1 | murrangatin | JLX4 |
| D-raffinose | DH1 | meranzin hydrate | JLX5 |
| stachyose | DH2 | demethylnobiletin | JLX6 |
| gluconic acid | DH3 | 6H-dibenzo(a,g)quinolizinium | LMZ1 |
| catalpol | DH4 | haplopine | LMZ10 |
| rehmannioside D | DH5 | γ-fagarine | LMZ11 |
| acteoside | E1 | dihydrochelerythrine | LMZ12 |
| 2-hydroxy-2-[2-oxo-2-[(2R,3R,4S,5R)-2,3,4,5-tetrahydroxy-6-oxohexoxy]ethyl]butanedioic acid | FL1 | nitidine | LMZ13 |
| umbelliferone | G1 | allocryptopine | LMZ14 |
| dictamine | H1 | uracil | LMZ15 |
| benzyl alcohol | HQ1 | magnoflorine | LMZ2 |
| baicalein | HQ10 | 5,7,8,15-tetrahydro-4-hydroxy-3-methoxy-6-methyl[1,3]benzodioxolo[5,6-e][2]benzazecin-14(6H)-one | LMZ3 |
| viscidulin III | HQ11 | vitexin | LMZ4 |
| dihydrobaicalein | HQ12 | ribalinine | LMZ5 |
| wogonin | HQ13 | 4H-dibenzo(de,g)quinolinium | LMZ6 |
| oroxylin A-7-o-beta-D-glucuronide | HQ14 | hesperidine | LMZ7 |
| 5-hydroxy-6-methoxy-2-phenyl-7-[3,4,5-trihydroxy-6-(hydroxymethyl)oxan-2-yl]oxychromen-4-one | HQ15 | norchelerythrine | LMZ8 |
| chrysin 7-glucuronide | HQ16 | sanguinarine | LMZ9 |
| 5,6-dihydroxy-2'-methoxy-7-(beta-D-glucurono pyranosyloxy)flavone | HQ17 | costunolide | MX1 |
| 6,8-dimethoxy-5-hydroxy-7-(beta-D-glucopyranuronosyloxy)-2-phenyl-4H-1-benzopyran-4-one | HQ18 | chlorogenic acid | MX2 |
| hispidulin | HQ19 | dehydrocostus lactone | MX3 |
| phenyl acetic acid | HQ2 | 7-methoxy-4-methyl-8-prop-2-enylchromen-2-one | NC1 |
| norwogonin | HQ20 | puerarin 4'-O-glucoside | NC2 |
| 5,7-dihydroxy-6,8,2',3'-tetramethoxyflavone | HQ21 | 2,6-bis(4-ethylphenyl)perhydro-1,3,5,7-tetraoxanaphth-4-ylethane-1,2-diol | NC3 |
| oroxylin A | HQ22 | oleamide | NC4 |
| 7,8-dihydroxyflavone | HQ23 | 1,2,3,4-tetramethylcyclopenta-1,3-diene | NC5 |
| darendoside A | HQ24 | 5,7-dihydroxy-2-phenyl-6,8-bis[3,4,5-trihydroxy-6-(hydroxymethyl)oxan-2-yl]chromen-4-one | NC6 |
| chrysin-6-C-glucoside-8-C-arabinoside | HQ25 | pachypodol | SCK1 |
| 5,7,2'-trihydroxy-6-methoxyflavone | HQ26 | dibutyl phthalate | SCK2 |
| 5,8-dihydroxy-6,7-dimethoxyflavone | HQ27 | marmesin | SCK3 |
| chrysin-6-C-arabinoside-8-C-glucoside | HQ3 | linoleic acid | SCK4 |
| scutellarin | HQ4 |  |  |

Table S2: Information of core targets.

| Common Name | Uniprot ID | BC | CC | Degree | San-Jiu-Wei-Tai granules (SJWTG) constituents acting on this target |
| --- | --- | --- | --- | --- | --- |
| SRC | P12931 | 0.131 | 0.490 | 49 | A1; A2; BS10; BS7; BS9; C1; HQ10; HQ11; HQ12; HQ13; HQ19; HQ20; HQ21; HQ26; JLX4; JLX5; JLX6; LMZ14; LMZ2; NC3; SCK1; SCK3 |
| STAT3 | P40763 | 0.156 | 0.500 | 49 | BS1; BS4; DH1; DH2; LMZ2 |
| MAPK3 | P27361 | 0.055 | 0.458 | 42 | HQ10; LMZ14; SCK4 |
| HSP90AA1 | P07900 | 0.124 | 0.474 | 41 | BS1; BS4; BS7; BS8; BS9; DH1; DH2; E1; HQ13; HQ24; HQ6; I1; JLX1; JLX4; JLX5 |
| MAPK1 | P28482 | 0.043 | 0.455 | 41 | C1; D1; LMZ14; LMZ5; LMZ8; NC3; SCK4 |
| AKT1 | P31749 | 0.062 | 0.449 | 36 | A1; BS10; G1; HQ11; HQ19; HQ21; HQ23; HQ26; HQ6; JLX4; JLX5; JLX6; LMZ10; LMZ12; SCK1 |
| HRAS | P01112 | 0.041 | 0.420 | 29 | BS7; BS8; BS9; LMZ7 |
| EGFR | P00533 | 0.029 | 0.431 | 27 | A1; A2; BS10; BS11; BS6; BS7; BS8; BS9; C1; G1; HQ10; HQ11; HQ13; HQ15; HQ16; HQ19; HQ20; HQ21; HQ22; HQ23; HQ26; HQ27; HQ4; HQ6; HQ8; HQ9; I4; I6; JLX2; JLX4; JLX5; JLX6; LMZ2; LMZ5; LMZ6; NC3; SCK1; SCK3 |
| JUN | P05412 | 0.065 | 0.444 | 27 | LMZ1 |
| IL6 | P05231 | 0.034 | 0.428 | 26 | SCK4 |
| NFKB1 | P19838 | 0.029 | 0.428 | 25 | G1 |
| VEGFA | P15692 | 0.018 | 0.422 | 24 | BS1; BS4; BS7; BS8; BS9; DH1; DH2; I1; NC3 |
| MAPK14 | Q16539 | 0.028 | 0.426 | 24 | H1; HQ17; JLX1; JLX4; JLX5; LMZ11; LMZ12; LMZ13; LMZ2; MX3; NC1; NC3; NC4; SCK2; SCK3; SCK4 |
| AR | P10275 | 0.054 | 0.443 | 22 | A1; A2; BS10; BS2; D1; HQ10; HQ13; HQ19; HQ20; HQ22; HQ23; HQ26; HQ27; HQ6; I5; I8; JLX4; LMZ14; LMZ2; LMZ3; LMZ5; MX1; MX3; NC1; SCK4 |
| TNF | P01375 | 0.027 | 0.400 | 21 | HQ14; HQ15; HQ16; HQ17; HQ18; HQ24; HQ4; HQ7; HQ8; HQ9; NC2 |
| MAPK8 | P45983 | 0.032 | 0.414 | 21 | H1; I2; JLX2; JLX4; LMZ11; LMZ12; LMZ14; LMZ5; NC3; SCK2; SCK3 |
| ERBB2 | P04626 | 0.018 | 0.406 | 17 | JLX2 |
